# The in vivo ISGylome links ISG15 to metabolic pathways and autophagy upon *Listeria monocytogenes* infection

**DOI:** 10.1038/s41467-019-13393-x

**Published:** 2019-11-26

**Authors:** Yifeng Zhang, Fabien Thery, Nicholas C. Wu, Emma K. Luhmann, Olivier Dussurget, Mariko Foecke, Clara Bredow, Daniel Jiménez-Fernández, Kevin Leandro, Antje Beling, Klaus-Peter Knobeloch, Francis Impens, Pascale Cossart, Lilliana Radoshevich

**Affiliations:** 1 Department of Microbiology and Immunology, University of Iowa Carver College of Medicine, Iowa City, IA 52242 USA; 2 Center for Medical Biotechnology, VIB, 9000 Gent, Belgium; 3 Department for Biomolecular Medicine, Gent University, 9000 Gent, Belgium; 4 Department of Integrative Structural and Computational Biology, The Scripps Research Institute, La Jolla, CA 92037 USA; 5 Institut Pasteur, Unité des Interactions Bactéries-Cellules, Département de Biologie Cellulaire et Infection, 75015 Paris, France; 6 Inserm, U604, 75015 Paris, France; 7 National Institute for Agronomic Research (INRA), Unité sous-contrat 2020, 75015 Paris, France; 8 Charité-Universitäts medizin Berlin, Corporate Member of Freie Universität Berlin, Humboldt-Universität zu Berlin, and Berlin Institute of Health (BIH), Institute of Biochemistry, Berlin, Germany; 9 Institute of Neuropathology, Medical Faculty, University of Freiburg, Freiburg, Germany; 10 Deutsches Zentrum für Herz-Kreislauf-Forschung (DZHK), Partner Site Berlin, Berlin, Germany; 11 VIB Proteomics Core, VIB, 9000 Gent, Belgium

**Keywords:** Proteomics, Autophagy, Infection, Bacterial host response, Post-translational modifications

## Abstract

ISG15 is an interferon-stimulated, ubiquitin-like protein, with anti-viral and anti-bacterial activity. Here, we map the endogenous in vivo ISGylome in the liver following *Listeria monocytogenes* infection by combining murine models of reduced or enhanced ISGylation with quantitative proteomics. Our method identifies 930 ISG15 sites in 434 proteins and also detects changes in the host ubiquitylome. The ISGylated targets are enriched in proteins which alter cellular metabolic processes, including upstream modulators of the catabolic and antibacterial pathway of autophagy. Computational analysis of substrate structures reveals that a number of ISG15 modifications occur at catalytic sites or dimerization interfaces of enzymes. Finally, we demonstrate that animals and cells with enhanced ISGylation have increased basal and infection-induced autophagy through the modification of mTOR, WIPI2, AMBRA1, and RAB7. Taken together, these findings ascribe a role of ISGylation to temporally reprogram organismal metabolism following infection through direct modification of a subset of enzymes in the liver.

## Abstract (web summary)

ISG15 is a ubiquitin-like modifier that can be upregulated in response to bacterial infections. Here, the authors use proteomics to identify endogenous ISGylation substrates in the liver of *Listeria monocytogenes* infected mice and show that ISGylation alters basal and infection-induced autophagy.

## Introduction

ISG15 is an interferon-induced, ubiquitin-like protein (ubl) with potent antiviral activity^1^. Similar to ubiquitin, ISG15 covalently modifies both cellular and viral substrates following activation by an E1 enzyme, UBE1L^2^, conjugation by an E2 enzyme, UBCH8^3^, and ligation by three identified E3 enzymes: HERC5/6, TRIM25, and HHARI^4–7^. USP18 is an ISG15-specific isopeptidase, which deconjugates ISG15 from its substrates^8^. However, USP18 is also a major negative regulator of STAT signaling, independent of its effect on ISGylation^9^. Canonical induction of ISG15 occurs following Type I interferon activation or viral infection; however, ISG15 can also be induced by a variety of other stimuli, such as retinoic acid, LPS, and genotoxic stress^10–12^. In addition, ISG15 can be upregulated in an interferon-independent manner following infection with bacterial pathogens, such as *Listeria monocytogenes* and *Mycobacterium tuberculosis*^13,14^. Murine models of ISG15 deficiency and studies in human cell lines have demonstrated that ISG15 and ISGylation broadly target numerous viruses^1,15^. *Isg15*-deficient animals develop normally, but are susceptible to both viral and bacterial pathogens upon challenge^11,13,15–17^. Furthermore, many viruses express ISG15 deconjugases or nonstructural proteins that prevent conjugation, suggesting evolutionary pressure to dismantle ISGylation for effective viral replication^18–20^. In this vein, an isopeptidase dead mutant of USP18^C61A/C61A^, which leads to enhanced ISGylation following ISG15 induction, is resistant to viral infection, while its susceptibility to bacterial infection is unknown^21^.

Despite the initial identification of ISG15 as an antiviral molecule, patients who lack *Isg15* do not appear to be overly sensitive to viral infection, instead they have difficulty clearing bacterial infections or the BCG vaccine due to reduced interferon gamma immunity^22^. Patients who lack ISG15 also develop an Aicardi-Goutières-like interferonopathy in the brain^23^. This was reported to originate from the destabilization of USP18, due to its role in STAT signaling. In addition, ISG15 functions through three distinct modes of action, which likely play complementary roles in various tissues following infection. ISG15 can be secreted and act as a cytokine^24,25^, has antiviral effects through non-covalent interactions^26^, and ISGylation can also reduce viral replication and viral load through covalent modification of viral and/or cellular targets^27,28^. In contrast to ubiquitin, the mechanistic consequences of ISGylation are not fully elucidated. However, several general themes have emerged regarding its function. ISG15 can modify nascent viral proteins co-translationally, thereby interfering with virion self-assembly^29^. ISGylation of cellular proteins alters trafficking, reducing both viral budding and exosome secretion, while increasing canonical cytokine secretion^13,30–32^. Finally, there are also several instances of ISGylation competing with ubiquitin to temporarily stabilize proteins that would otherwise be degraded^33^.

In order to better understand the consequences of ISG15 modification on protein fate, we systematically identified endogenous ISG15 substrates following infection in vivo with the bacterial pathogen, *Listeria monocytogenes*. Enrichment of peptide remnants left by posttranslational modifications after trypsin digestion coupled with identification of the precise site of modification through mass spectrometry has been extremely fruitful in unraveling mechanisms of action of other modifications, such as acetylation^34^, ubiquitination^35–37^, and SUMOylation^38–40^. While several studies have identified ISGylated targets through proteomics^41–44^, these approaches primarily relied either on ectopic expression of ISG15 in tissue culture cells, which could lead to overexpression artifacts, or interferon treatment of primary cells, which could deliver a stronger stimulus than that which is physiologically induced by a viral or bacterial infection. It has been challenging to differentiate between ISG15, ubiquitin, and NEDD8 sites because these UBLs share the same diglycine adduct following digestion with trypsin. A recent study used a distinct immuno-enrichment strategy to purify ubiquitin sites in vitro^45^. However, thus far no one has been able to specifically identify ISGylated sites either in vitro or in vivo.

Here, we address this obstacle by defining the quantitative, proteome-wide ubiquitylome in *Isg15*-deficient animals prior to and following infection. We highlight sites that arise during *Listeria* infection in wild-type animals, as well as in mice with enhanced ISGylation due to knock in of an inactive ISG15 deconjugase (USP18^C61A/C61A^). Our strategy reveals 930 endogenous sites on 434 ISG15 substrates in the liver in the context of a clinically relevant infection. Unlike for SUMOylation, there is neither a discernable consensus motif for modification nor an enrichment of modification sites in a specific cellular compartment. Interestingly, ISG15 modifies metabolic enzymes on lysines at dimerization interfaces and catalytic sites in the liver. In particular, ISGylation targets four key regulators of autophagy and leads to upregulation of this pathway following infection both in vivo and in vitro.

## Results

### Strategy for Identification of bona fide ISG15 sites

**Fig. 1:**
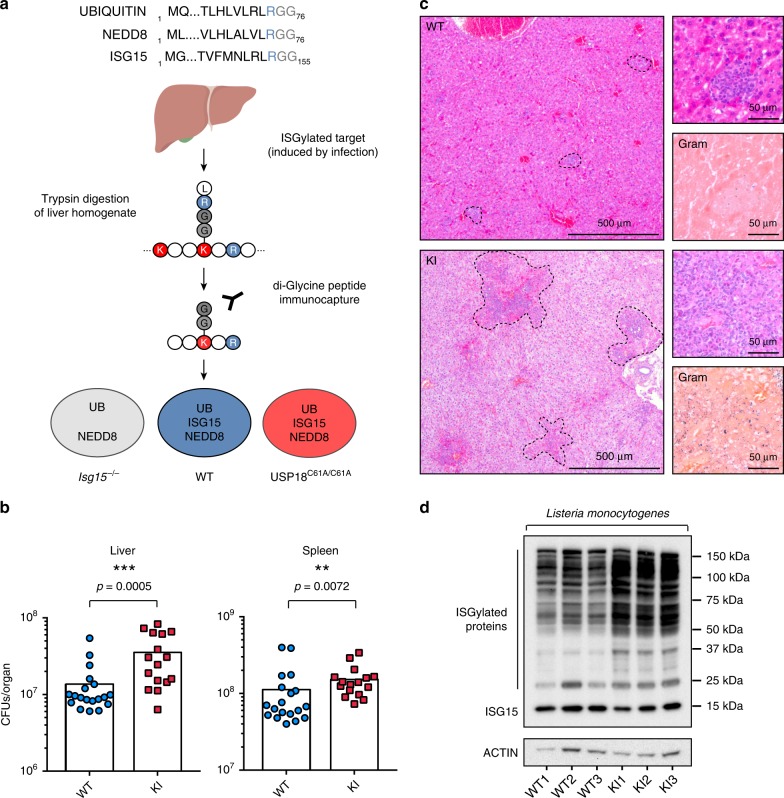
Strategy for Identification of ISG15 sites and the effect of enhanced ISGylation on bacterial infection. **a** C-terminal amino acid sequence of Ubiquitin, ISG15, and NEDD8 followed by the experimental design of proteomic identification of ISG15 diglycine sites. Created with Biorender.com. **b** Colony-forming units of *Listeria monocytogenes* per organ following 72 h of infection (5 × 10^5^ colony-forming units injected intravenously into the tail vein); the results displayed are from three independent experiments, bar indicates mean, wild-type *n* = 18, USP18^C61A/C61A^ *n* = 16 (two KI animals died and thus CFUs were not enumerated) significance for in vivo data determined using Mann–Whitney test. **c** Representative hemotoxylin and eosin and Gram staining of liver tissue of animals infected with *Listeria monocytogenes* for 72 h (wild-type *n* = 3 and USP18^C61A/C61A^ *n* = 3). Bacterial foci are indicated by black dotted line. **d** SDS-PAGE of liver homogenates from wild-type or USP18^C61A/C61A^ mice following 72 h of infection (representative blot with *n* = 3 animals per genotype) with *Listeria monocytogenes*.

In order to elucidate mechanisms of action of ISG15, we used mass spectrometry (MS)-based proteomics to identify ISG15 modification sites in an unbiased manner. We analyzed liver tissue from mice infected with *Listeria monocytogenes* and enriched peptides that carry a diglycine adduct following digestion with trypsin. To distinguish bona fide ISGylation sites from ubiquitin sites, which share a conserved C-terminal LRLRGG motif (Fig. 1a), we compared wild-type (WT) mice with *Isg15-*deficient mice (KO). In addition, we mapped sites in mice bearing a knock-in (KI) mutation in USP18^C61A/C61A^, which renders it catalytically inactive, leading to hyper-ISGylation following infection, a condition which is biologically interesting in addition to being a positive control for our screen (Fig. 1a). USP18 has two distinct mechanisms of action: (i) it removes ISG15 from substrates and (ii) it negatively regulates STAT signaling^9^. Since USP18 deletion cannot distinguish between these functions, we first assessed whether the deconjugase-dead (USP18^C61A/C61A^) mouse would be resistant to *Listeria* infection. USP18^C61A/C61A^ mice exhibit increased resistance to viral infection, but are otherwise phenotypically indistinguishable from wild-type littermates^21^. However, surprisingly *Listeria* infection of USP18^C61A/C61A^ mice leads to a significant increase in bacterial load in the liver relative to wild-type controls (Fig. 1b). While there was also a slight but significant increase in bacterial burden in the spleen in USP18^C61A/C61A^ animals, the bacterial counts in the liver indicated an unusual susceptibility in that organ. Histological sections of infected liver revealed larger more diffuse bacterial foci which penetrated into neighboring hepatocytes in USP18^C61A/C61A^ animals, whereas in wild-type animals the bacteria were primarily contained within immune cells (Fig. 1c). In addition, Gram staining confirmed the presence of many more bacteria per focus in USP18^C61A/C61A^, relative to wild-type animals, as the CFUs per organ indicated (Fig. 1c). Accordingly, the liver tissue from infected USP18^C61A/C61A^ mice also displayed enhanced ISGylation compared with the WT (Fig. 1d). Together, these data indicate a tissue-specific role for ISGylation in the liver upon *Listeria* infection, and provided a rationale to map ISG15 sites in the liver of USP18^C61A/C61A^ animals compared with wild-type animals.

**Fig. 2:**
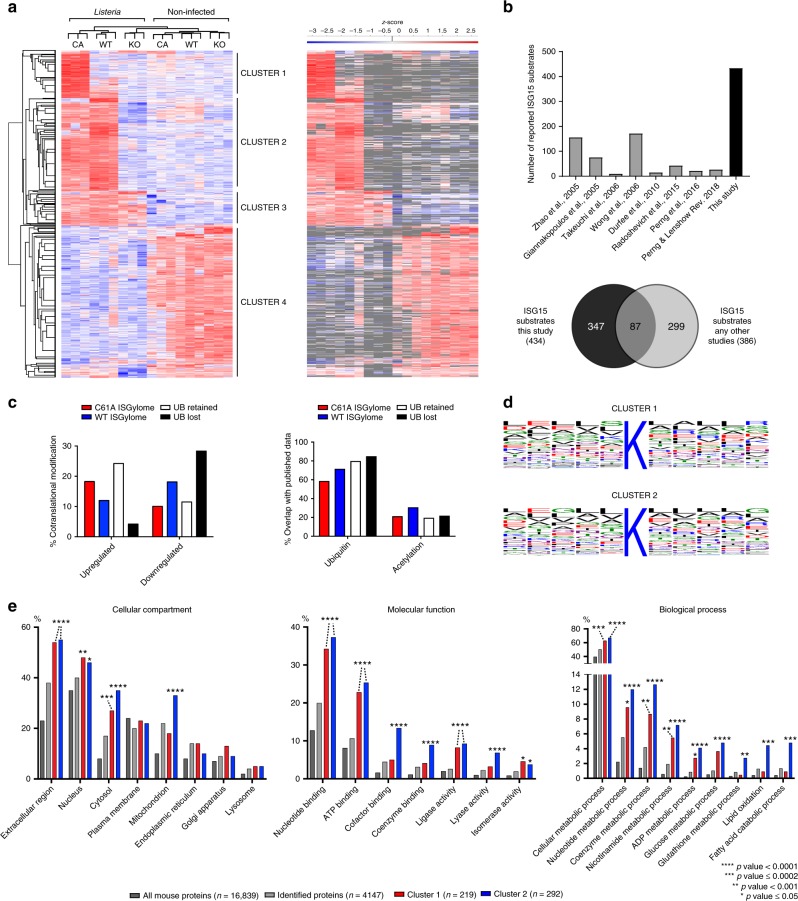
Identification of in vivo ISGylation sites during *Listeria* infection. **a** Heatmap showing significantly regulated GlyGly(K) sites after non-supervised hierarchical clustering. On the right side, the heatmap is shown with missing values in gray. Four major clusters can be observed corresponding to ISG15 sites (clusters 1 and 2) and ubiquitin sites (clusters 3 and 4). **b** Bar chart and Venn diagram showing the number and overlap of ISGylated proteins identified in this study and previous studies. **c** Percent of modified proteins from each cluster that are also upregulated following infection according to the shotgun data from liver tissue prior to diglycine enrichment; Percent of modified sites from each cluster that overlap with published ubiquitin or acetylation sites. **d** Sequence logos were drawn based on the sequence alignment of different ISGylation sites and their + 5 and −5 flanking regions. **e** GO analysis of ISGylated proteins (clusters 1 and 2) relative to all proteins identified in this analysis (gray) and all mouse proteins in the Uniprot/Swiss-Prot database (black). Bars correspond to the percentage of proteins annotated with each GO term. Asterisks indicate significant enrichment relative to the number of identified proteins (two-sided Fisher's exact test).

Consequently, for the proteomics analysis, we infected *Isg15*^−/−^, wild-type, and USP18 ^C61A/C61A^ mice via intravenous injection and harvested liver tissue following 72 h of infection. We also included a noninfected cohort. Upon trypsin digestion and enrichment of diglycine adducts from the liver, diglycine-modified peptides were identified and quantified. Following statistical analysis and non-supervised hierarchical clustering, significantly regulated sites grouped into four major clusters. Moreover, animals also clustered together by genotype and condition, indicating the high reproducibility of our approach (Fig. 2a). Prior to infection, ~1000 ubiquitin sites were identified across all three conditions (cluster 4). *Listeria* infection led to a global downregulation of these ubiquitin sites, as previously observed for both ubiquitin and SUMO modification in vitro^38,46^. We also identified 219 ubiquitin sites that arise following infection (cluster 3). Most importantly, we were able to identify 614 ISG15 sites on 292 proteins induced by *Listeria* infection (cluster 2). The majority of these sites are completely absent in *Isg15*^−/−^-deficient animals as indicated in gray (Fig. 2a, right panel), and are therefore considered bona fide ISG15 sites. We also identified 316 ISG15 sites on 219 proteins that were primarily present in USP18^C61A/C61A^ animals (cluster 1). Since USP18 is concomitantly induced with ISG15 following *Listeria* infection, we suspect that certain substrates are deconjugated more rapidly than others. Future work should address this hypothesis by site identification during a time course. To avoid mistakenly identifying ubiquitin or NEDD8 sites as ISG15 sites (due to possible indirect effects of *Isg15* deletion or USP18^C61A/C61A^ mutation), we assessed whether ubiquitin and NEDD8 levels appear to vary using SDS-PAGE prior to or following infection (Supplementary Fig. 1a, b). If anything, ubiquitin and NEDD8 sites decreased following infection in vivo while ISGylation increased, as was previously observed for ubiquitin^46^ and as is a new observation for NEDD8 (Supplementary Figs. 1b, 2e). In vitro total ubiquitin did not dramatically change in mouse embryonic fibroblasts 24 h post infection, whereas NEDD8 was slightly reduced (Supplementary Fig. 1e). Furthermore, a comparison of sites identified by quantitative diglycine enrichment between *Isg15*^−/−^ and wild-type animals prior to infection shows no significantly regulated sites (Supplementary Fig. 1g). Taken together, these data suggest that sites identified by our method are likely bone fide ISG15 sites and by extension not misidentified ubiquitin or NEDD8 sites. Ultimately, we identified 930 ISG15 sites on 434 proteins (clusters 1 and 2, Supplementary Data 1), 87 of which had been previously identified (Fig. 2b; Supplementary Data 2). Taken together, our approach systematically identified endogenous ISG15 modification sites in vivo.

### ISG15 can modify known ubiquitin and acetylation sites

Analysis of the primary amino acid sequences of identified ISG15 sites did not reveal an obvious amino acid consensus sequence for the modification (Fig. 2d), as is the case for ubiquitin^36,47^, but unlike for SUMO^40,48^. One proposed mode of action of ISG15 is cotranslational modification of nascent polypeptides following viral infection as mediated by the IFN-induced E3 HERC5^29^. Since *Listeria* can provoke ISG15 induction in an interferon-independent manner, we assessed whether there was a potential bias toward ISGylation of proteins induced by infection, which could correlate with cotranslational modification, as determined by quantitative proteomics of the input (Supplementary Fig. 2a, Supplementary Data 3). Comparison with ubiquitinated proteins suggested a lower percentage of modification of infection-induced ISG15 targets than that of infection-induced ubiquitin targets (Fig. 2c). It will be worthwhile to mechanistically determine whether *Listeria* infection leads to similar or reduced levels of cotranslational modification relative to viral infection in future studies.

To date, over 100,000 modification sites have been identified for ubiquitin; however, one important caveat of these diglycine enrichment-based studies is that they did not exclude ISG15 and NEDD8 sites. We therefore compared published ubiquitin sites^49^ with our identified ISG15 sites (Fig. 2c) in order to assess the overlap of sites, which could arise from competition between ubiquitin and ISG15 or misidentified ubiquitin sites. First, sites identified prior to infection in our screen in *Isg15*^−/−^ animals (and therefore expected ubiquitin sites) had an 84% overlap with published ubiquitin sites. Ubiquitin sites induced by infection (present in *Isg15*-deficient animals) had a 75% overlap with published ubiquitin sites. Notably, sites identified in the condition of deregulated ISGylation had only a 57% overlap with ubiquitin sites, whereas those sites which arose in wild-type animals following *Listeria* infection had a 71% overlap. Future biochemical analysis of site competition between distinct posttranslational modifications on specific substrates will be critical to tease apart distinct functions of ubiquitin and ISG15 under these conditions.

We also compared ISG15 sites with identified acetylation sites. The percentage of common sites between ISG15 and acetylation in clusters 1, 3, and 4 was similar (21%, 19%, and 21% of sites, respectively). Whereas ISG15 sites induced following infection with *Listeria* in wild-type mice (cluster 2) have a 31% overlap with acetylation. Previous work has indicated that acetylation preferentially targets the metabolic activity of enzymes in the liver^50^. While acetylation of specific enzymes can have distinct effects depending on the enzyme, it is tempting to speculate that ISGylation could compete for lysines on enzymes that would otherwise be activated by acetylation during infection to thwart bacterial acquisition of host nutrients. Taken together, ISGylation following *Listeria* infection shows significant overlap with both ubiquitination and acetylation, suggesting competition for specific lysines.

### ISG15 modifies extracellular and mitochondrial proteins

Gene ontology analysis of ISGylated proteins for cellular compartments highlighted a significant enrichment in extracellular proteins in all infection conditions (Fig. 2e). These results support the published role for ISGylation in exosome secretion, as well as the finding that ISGylation modulates cytokine secretion, and highlight other potential substrates in addition to TSG101 that could potentially affect this phenotype^13,32,51^. The identified ISGylome following *Listeria* infection in wild-type animals was significantly enriched in mitochondrial proteins in liver tissue (Fig. 2c). Recent work has indicated that ISG15 can affect mitochondrial morphology and function following Vaccinia virus infection; our identification of an enrichment of mitochondrial proteins that are ISGylated following *Listeria* infection is in concordance with these findings^52^. Gene ontology analysis of ISGylated proteins for molecular function revealed an enrichment of proteins that bind to nucleotides and ATP in both USP18^C61A/C61A^ and wild-type conditions (Fig. 2e). Following infection of wild-type animals, there is an enrichment of ISGylation of cofactor and coenzyme-binding proteins as well as lyase activity, which is absent in animals with enhanced ISGylation. In addition, gene ontology analysis of ISGylated proteins for biological processes revealed a striking enrichment in a number of different metabolic pathways, including ADP, glutathione, and glucose metabolism as well as lipid oxidation (Fig. 2e). Taken together, ISG15 modifies a number of proteins in the liver, which are relevant for the control of metabolism.

### Evidence for ISG15 chains or ISG15/ubiquitin chains

**Fig. 3:**
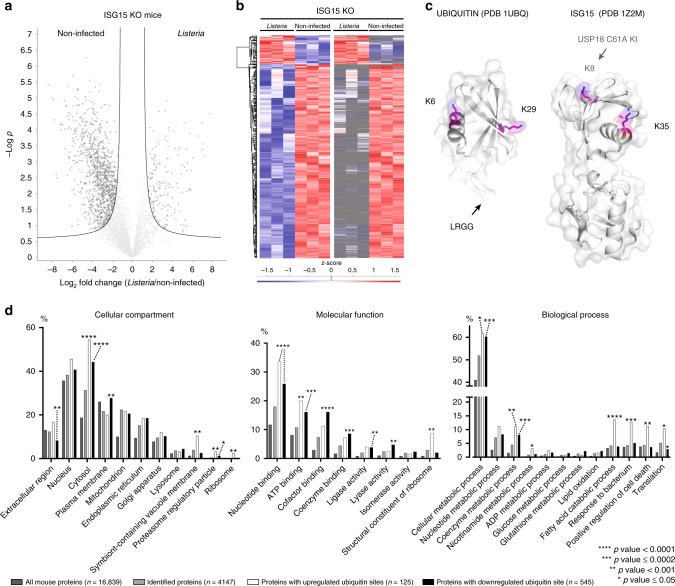
Loss of ubiquitin sites following infection and evidence for mixed ISG15/Ubiquitin chains. **a** Volcano plot showing significantly regulated ubiquitin sites upon *Listeria* infection of *Isg15*^−/−^ animals. The fold change (in log2) of each GlyGly(K) site is shown on the *x*-axis, while the statistical significance (−log *P*-value) is shown on the *y*-axis. In total, 168 GlyGly(K) sites were significantly upregulated during infection, while 1138 sites were downregulated (Supplementary Data 3). **b** Heatmap representation of significantly up- and downregulated ubiquitin sites after non-supervised hierarchical clustering. On the right side, the heatmap is shown with missing values in gray (Supplementary Data 4). **c** Crystal structures of ubiquitin and ISG15 with modified lysines highlighted. K6 and K29 of ubiquitin are modified following bacterial infection in wild-type animals. K35 of ISG15 is modified following bacterial infection in wild-type animals, and K8 is modified following infection in USP18^C61A/C61A^ animals. **d** GO cellular compartment analysis of ubiquitylated proteins (clusters 3 and 4) relative to all proteins identified in the analysis (light gray) and all mouse proteins in the Uniprot/Swiss-Prot database (dark gray). Bars correspond to the percentage of proteins annotated with each GO term. Asterisks indicate significant enrichment relative to the number of identified proteins (two-sided Fisher's exact test).

While our primary goal was to identify ISG15 sites, determining the strict ubiquitylome in *Isg15*^−/−^animals allowed us to gain insight into how ISG15 and ubiquitin modifications cooperate during an infection. Indeed, it was immediately apparent that a large number of ubiquitin sites (cluster 4) disappeared following infection and that others emerged, likely following transcriptional upregulation of the ubiquitinated substrate (Fig. 3a, b; Supplementary Fig. 1a, Supplementary Data 4). Interestingly, we also found evidence for modification of Ubiquitin on K6 and K29 and modification of ISG15 on K35 in wild-type infection conditions and on K8 in USP18^C61A/C61A^ KI mice (Fig. 3c). Our analysis cannot formally differentiate between modification by ubiquitin and ISG15, since ISG15 is absent in the strict ubiquitylome by design, however, these data do confirm that K29 of ubiquitin is modified. Mixed chains between ubiquitin and ISG15 have previously been identified on this lysine^53^. Modification of ISG15 on K8 or K35 (Fig. 3c) has not yet been described so whether this represents mixed chains of ubiquitin and ISG15 or ISG15*-*ISG15 chains remains to be determined. Future biochemical studies of the consequences and nature of these chains following infection will likely be worthwhile.

Gene ontology analysis of ubiquitin sites gained and lost following infection (clusters 3 and 4, respectively) revealed that certain substrates are similarly enriched among ubiquitin targets and ISG15 sites (nucleotide binding and cellular metabolic processes), however, others inversely correlated with ISG15 sites (cofactor binding, coenzyme binding, lyase and ligase activity, Fig. 3d). These patterns of enrichment suggest that certain ubiquitin modifications are replaced with ISG15 modifications following infection. Interestingly, ISG15 sites on proteins related to metabolic processes are not significantly enriched among ubiquitin sites, suggesting that ISGylation specifically targets proteins that affect metabolism. Whereas, for ubiquitin, there are several groups of substrates associated with the response to pathogens, cell death and translation, all of which further differentiate ubiquitin sites from ISG15 sites.

### Structural analysis of ISG15 sites

**Fig. 4:**
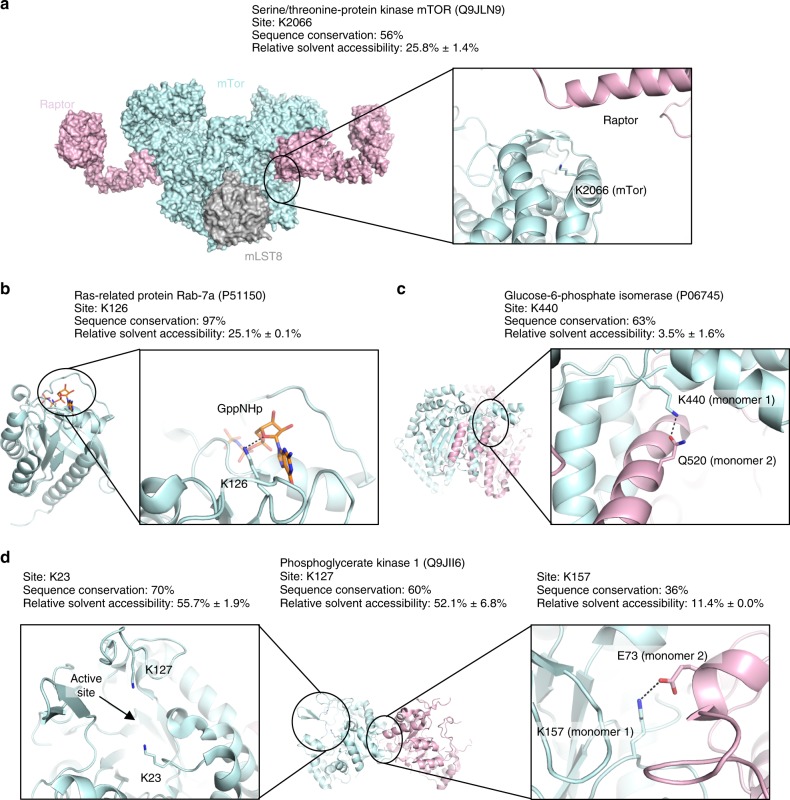
Structural analysis of ISGylated sites. **a** ISGylated site Lys2066 of serine/threonine-protein kinase mTOR (uniprot ID: Q9JLN9) is shown on the structure PDB 5H64 [https://www.rcsb.org/structure/5H64]^73^. **b** ISGylated site Lys126 of Ras-related protein Rab-7a (uniprot ID: P51150) is shown on the structure PDB 1VG8 [https://www.rcsb.org/structure/1VG8]^74^. **c** ISGylated site Lys440 of glucose-6-phosphate isomerase (uniprot ID: P06745) is shown on the structure PDB 2CVP [https://www.rcsb.org/structure/2CVP]^75^. **d** ISGylated sites Lys23, Lys127, and Lys157 of phosphoglycerate kinase 1 (uniprot ID: Q9JII6) is shown on the structure PDB 4GAC [https://www.rcsb.org/structure/4GAC]^76^. **a**–**d** Relative solvent accessibility (RSA) is computed using all available structures. The mean and the standard deviation are reported. Hydrogen bonds and electrostatic interactions are represented by black dashed lines.

While there was no discernable primary amino acid sequence motif for ISG15 sites, we hypothesized that there might be specific domains or structural features targeted by the UBL. We therefore focused on ISG15 sites in proteins with known crystal structures to search for common features surrounding the identified target lysines. In several cases, ISG15 modification occurs on sites of protein/protein interactions, which can be between several proteins in a complex (Fig. 4a) or at dimerization interfaces (Fig. 4b). In fact, the mammalian target of rapamycin (mTOR) is modified on lysine 2066 following infection with *Listeria* in wild-type animals, within the FKBP rapamycin-binding domain^54^. mTOR is not only critical for the immune response to pathogens but also negatively regulates the catabolic process of autophagy, which can target bacteria for degradation in the lysosome. A second autophagy regulator, the Ras-related protein RAB7A, also emerged from our analysis of ISG15 targets. RAB7A is modified by ISG15 in the condition of deregulated autophagy (USP18^C61A/C61A^ KI mice). Notably, RAB7A is modified in close proximity to its GTP-binding domain at K126. In other cases, ISGylation occurs near GTP-binding sites or active sites of enzymes (Fig. 4b, d). Modification with a bulky di-ubiquitin-like molecule at these sites could potentially hinder enzymatic activity or GTPase function. While the proximity of target lysines to these domains was not significantly closer than that of other lysines on the same structure (Supplementary Fig. 3a), our systematic gene ontology analysis revealed an enrichment in nucleotide and ATP-binding proteins, as well as cofactor and coenzyme-binding proteins (Fig. 2e, Molecular function panel). In addition, many metabolic enzymes such as glucose-6-phosphate isomerase and phosphoglycerate kinase 1 are also modified by ISG15 following infection with *Listeria* either at dimerization domains or at active sites of the enzyme (Fig. 4c, d). Taken together, ISGylation of liver enzymes, modulators of growth factor signaling, and catabolic pathways, suggests a new role for ISGylation in liver metabolism during infection.

### ISGylation increases basal and infection-induced autophagy

**Fig. 5:**
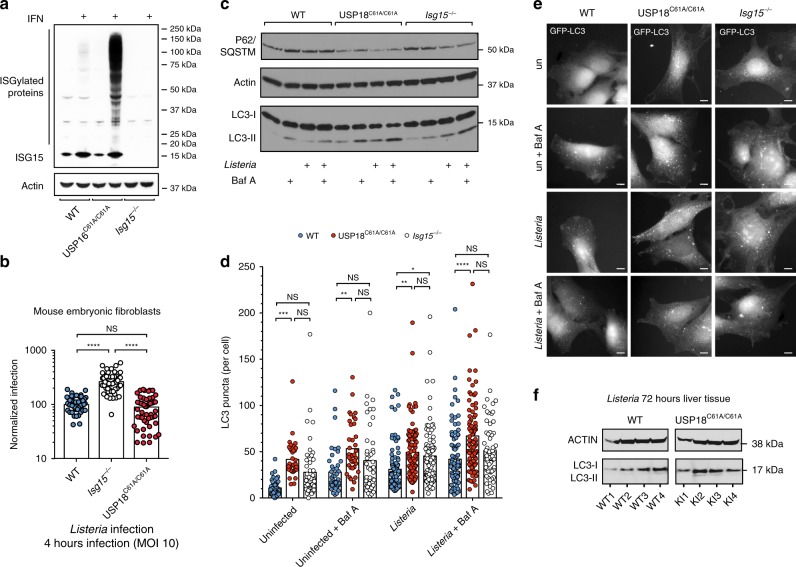
ISGylation alters basal and infection-induced autophagy. **a** SDS-PAGE of wild-type, USP18^C61A/C61A^, and *Isg15*^−/−^ mouse embryonic fibroblasts treated with interferon β (1000 units/mL for 24 h), bottom ISG15 blot is a short exposure of the upper panel. **b** Mouse embryonic fibroblasts MEFs (wild-type, *Isg15*^−/−^ KO, and USP18^C61A/C61A^ KI) were infected with *Listeria monocytogenes* strain EGD for 4 h at an MOI of 10. Cells were lysed, and bacterial CFUs were enumerated through serial dilution. Wild-type values were normalized to 100; data from three independent experiments are shown. Statistical analysis was conducted using one-way ANOVA and Tukey’s test post hoc. **c** SDS-PAGE analysis of wild-type or USP18^C61A/C61A^ MEFs treated with Bafilomycin A (100 nM for 20 min prior to lysis) at baseline or following 4 h of infection with *Listeria* at an MOI of 10. Immunoblot is a representative image of five independent experiments. **d** GFP-LC3 puncta per cell; data shown are compiled from four separate experiments WT (un, un + Baf, EGD, EGD + Baf) *n* = 408, 450, 984, 987 (USP18^C61A/C61A^ (un, un + Baf, EGD, EGD + Baf) *n* = 352, 410, 1038, 1014, *Isg15*^−/−^ (un, un + Baf, EGD, EGD + Baf) *n* = 183, 224, 710, and 470 individual cells per condition were enumerated). Statistical analysis was conducted using one-way ANOVA and Tukey’s test post hoc (exact *p*-values (Supplementary Fig. 4b). NS nonsignificant, *p < 0.05; **p < 0.01; ***p < 0.001 ****p < 0.0001. **e** Representative images of cells stably expressing GFP-LC3 and treated as above with Bafilomycin A or infected with *Listeria* and treated with Bafilomycin A, scale bar is 5 μm. **f** SDS-PAGE of liver homogenate from *n* = 4 individual animals either wild-type or USP18^C61A/C61A^ infected with 5 × 10^5^ Listeria, organs harvested following 72 h of infection. Raw data are available in the source data file.

We were struck by the modification of four key regulators of autophagy by ISGylation, in particular the modification of mTOR and WIPI2 following infection in wild-type animals following *Listeria* infection and modification of AMBRA1 and RAB7 following infection in USP18^C61A/C61A^ animals. Given the particular relevance of these proteins in the control of autophagy, we explored whether this pathway is altered by ISG15 upon *Listeria* infection. Autophagy is a catabolic, cellular response to starvation, but it can also control targeting of intracellular pathogens to the lysosome for degradation or indirectly alter a pathogen’s access to cytosolic nutrients required for growth and replication. *Listeria* expresses ActA to evade autophagy as it spreads from cell-to-cell^55^. Recent work suggests that if ActA levels plunge, the bacterium can be trapped and replicate in a vacuole in hepatocytes in a process that could potentially involve autophagic targeting^56^. We reasoned that either of these pathways could be affected by altered autophagy induction in USP18^C61A/C61A^ animals or cells, where ISG15 modification is enhanced. In order to address this question, we generated mouse embryonic fibroblasts (MEFs) from wild-type, USP18^C61A/C61A^, or *Isg15*^−/−^ animals. We first assessed ISGylation levels in wild-type versus USP18^C61A/C61A^ cells following interferon treatment and found that levels of ISGylation in USP18^C61A/C61A^ MEFs relative to wild-type are much higher as expected (Fig. 5a). Surprisingly, longer exposure using film revealed that there is increased ISGylation prior to interferon treatment as well (Supplementary Fig. 4a). We took advantage of this unexpected increased ISGylation in fibroblasts to mimic in vivo ISGylation following *Listeria* infection. Hyper-ISGylation is as protective against *Listeria* infection as wild-type ISGylation in tissue culture cells in contrast to *Isg15*-deficient fibroblasts, which are highly susceptible to *Listeria* infection (Fig. 5b). In fact, upon normalization of bacterial load across experiments, there is evidence for a bimodal distribution, suggesting that in a subset of cells hyper-ISGylation can be more protective than wild-type ISGylation in a cell intrinsic manner similar to what was reported for viral pathogens^21^. These findings support our previous work that demonstrates that overexpression of ISG15 protects against *Listeria* infection in non-phagocytic cells^13^. We next sought to quantify autophagy in these cells by assessing endogenous conversion of the autophagic marker LC3-I to LC3-II via SDS-PAGE analysis (Fig. 5c). Autophagy was dramatically upregulated in USP18^C61A/C61A^ fibroblasts relative to wild-type cells prior to infection at baseline, and autophagic flux was assessed by the addition of Bafilomycin A which inhibits lysosomal acidification. Infection with *Listeria* induced autophagy in both wild-type and USP18^C61A/C61A^ cells, however, this induction was dramatically and significantly increased in cells with enhanced ISGylation (Fig. 5c). Furthermore, the autophagy adaptor p62/SQSTM, which is also targeted for degradation by autophagy, was reduced in quantity in USP18^C61A/C61A^ MEFs relative to wild-type MEFs at baseline and was further degraded following *Listeria* infection; this degradation could be partially but not completely halted by Bafilomycin A treatment (Fig. 5c). Interestingly, ISG15 deletion also increases autophagy, as was previously observed^57^, however, p62 accumulates under these conditions, thus it seems that targeting of autophagosomes to the lysosome may be impaired in *Isg15*-deficient cells (Fig. 5c). We subsequently ectopically expressed an autophagic marker, GFP-LC3, in wild-type, USP18^C61A/C61A^ and *Isg15*^−/−^ fibroblasts and quantified basal autophagy, as well as autophagy following infection by enumerating LC3 puncta (Fig. 5d, e). Across all conditions, there were significantly more LC3 puncta per cell in USP18^C61A/C61A^ fibroblasts than in wild-type cells, whereas *Isg15*^−/−^ cells displayed an intermediate phenotype that was not significantly different from either wild-type or USP18^C61A/C61A^ cells, and did not increase in Bafilomycin A treated cells supporting an impairment in trafficking. Finally, we wondered whether the effect on autophagy induction would also occur in vivo following *Listeria* infection. We assessed LC3-II levels in liver homogenate isolated from wild-type and USP18^C61A/C61A^ animals following 72 h of infection and found increased LC3-I as well as LC3-II in USP18^C61A/C61A^ animals (Fig. 5f). Taken together, as predicted by our global ISGylome analysis, modification of key modulators of autophagy led to increased basal and infection-induced activation of this pathway in both cells and animals with enhanced ISGylation.

**Fig. 6:**
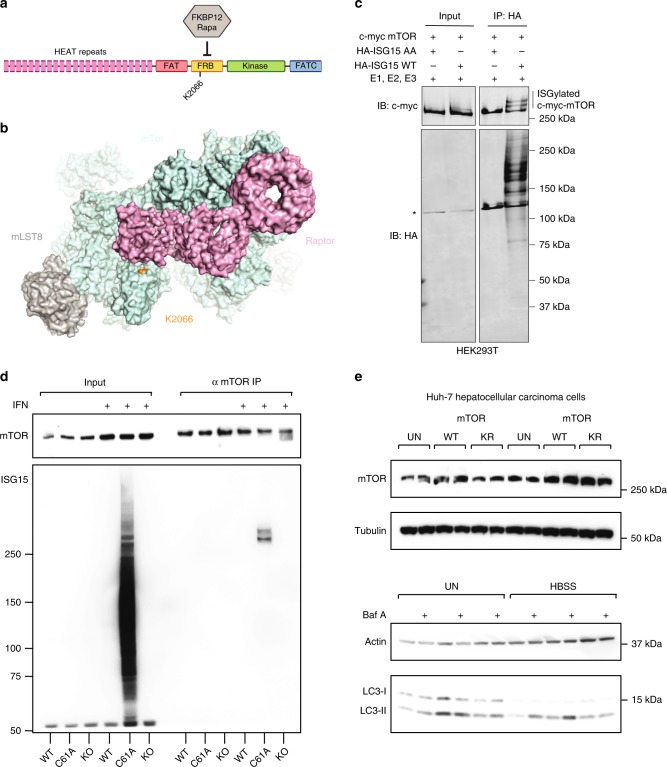
mTOR is modified by ISG15 and mutation of K2066 to arginine results in reduced autophagy. **a** Schematic of the domains of mTOR with the target lysine highlighted. **b** ISGylated site Lys2066 of serine/threonine-protein kinase mTOR (uniprot ID: Q9JLN9) is shown in orange on the structure PDB 5H64 [https://www.rcsb.org/structure/5H64]^73^. **c** Validation of modification of mTOR by ISG15 in HEK293T overexpression system (simultaneous overexpression of E1, E2, E3, mTOR, and ISG15, either wild-type or non-conjugatable ISG15AA); asterisk indicates FLAG-HERC5; SDS-PAGE of input and immunoprecipitation of ISG15. **d** Validation of endogenous modification of mTOR by ISG15 in mouse embryonic fibroblasts treated with Type I IFN (1000 units/mL) for 24 h. Immunoprecipitation of endogenous mTOR and SDS-PAGE for both mTOR and ISG15. Modified ISG15 is only detectable in USP18^C61A/C61A^ MEFs. The experiment was repeated three times, and one representative blot is shown. **e** Ectopic expression of either wild-type mTOR (WT) or mTOR K2066R (KR) in Huh-7 liver cells untreated or HBSS starved (4H) and/or treated with Bafilomycin A (100 nM for 30 min prior to lysis). The experiment was repeated three times, and one representative blot is shown. Raw data are available in the source data file.

In order to determine whether ISG15 modification of autophagy regulators has a direct effect on LC3 turnover, we chose to focus on mTOR since it plays such a critical role in growth and metabolism. ISG15 targets K2066 of mTOR which lies in the FKPB/Rapamycin-binding domain (FRB) (Fig. 6a, b). In order to validate modification of mTOR by ISG15, we transfected HEK293T cells with ISG15, its E1, E2, E3, and mTOR^58^ (Fig. 6c). ISGylated mTOR was visible following transfection and immunoprecipitation of wild-type ISG15, but not with a mutant of ISG15 that is unable to conjugate (ISG15AA). In addition, we validated modification of Myosin 9^59^, HSP90^60^, and glucose-6-phosphate isomerase 1 (mGPI-1) (Supplementary Fig. 5a–c). Since overexpression in this system is reported to lead to promiscuous cotranslational modification of accessible lysines^29^, we endeavored to validate endogenous modification of mTOR in USP18^C61A/C61A^ MEFs that were treated with IFN as a model for in vivo infection. Upon immunoprecipitation of endogenous mTOR, a clear doublet corresponding to ISG15 is present in the USP18^C61A/C61A^, but not in *Isg15*^−/−^ or wild-type MEFs (Fig. 6d). Probing immunoprecipitated mTOR with antibodies against ubiquitin or NEDD8 did not reveal modification by these proteins, which further validates the identification of bone fide ISG15 sites by our approach (Supplementary Fig. 5e–g). The doublet could represent two individual mono-ISGylation sites or a chain of ISG15 molecules given our observation of sites on ISG15 itself. Since we only quantified one modification site on mTOR, we mutated this lysine, K2066, to arginine and assessed the effect of this mutation on autophagy induction in a human hepatocellular carcinoma cells Huh-7 (Fig. 6e). Amino acid starvation of these cells led to an increase in LC3-II as expected, ectopic expression of mTOR K2066R induced relatively less LC3-II than wild-type mTOR or mock transfected cells, indicating that ISGylation of mTOR could repress its activity. This observation, however, was made in cells that harbor endogenous mTOR, therefore future work to assess the effect of a gene-edited KR mutant in the absence of wild-type mTOR as well as in other ISG15 substrates from our analysis will be of interest to mechanistically assess the effect of ISGylation on specific target lysines. Taken together, mTOR is ISGylated on lysine K2066 in the liver of *Listeria*-infected animals, we validated that this modification occurs in vitro in both human and mouse cells and mutating lysine 2066 to arginine has a repressive effect on the downstream process of autophagy in an overexpression model in human liver cells.

## Discussion

Here, we have devised a strategy combining murine models of ISG15 deficiency and enhanced ISGylation with quantitative label-free proteomics to map the endogenous liver ISGylome in animals infected with the bacterial pathogen, *Listeria monocytogenes*. We have identified and distinguished bona fide ISGylated sites from ubiquitin sites in vivo. We have mapped 930 ISG15 sites on 434 proteins, which is a substantial increase from previous studies of ISG15 conjugation. In addition, we have observed that ISG15 modification occurs at known acetylation sites following infection, and on extracellular/secreted and mitochondrial proteins. We have observed that ISG15 modification targets metabolic pathways and through a computational analysis of available structures of ISG15 target proteins have highlighted that ISG15 often modifies sites of protein dimerization or catalytic sites of enzymes. We validated our findings by assessing the role of modification of known regulators of the catabolic process of autophagy and found that basal and infection-induced autophagy were increased in cells and animals with enhanced ISGylation (USP18^C61A/C61A^).

The enrichment of adducts of specific posttranslational modifications left following trypsin digestion has revolutionized the understanding of the ubiquitin code, the acetylome, and helped to systematically identify the SUMOylome as well^61^. ISG15 has been challenging to study due to its absence in genetically tractable model organisms such as yeast, its multiple modes of action (as a cytokine, covalent modification and non-covalent interaction), its absence prior to induction following infection and its C-terminal LRLRGG motif, which is indistinguishable from that of ubiquitin or NEDD8. Initial identification of ISG15 substrates following ectopic expression, enrichment, and affinity purification suggested widespread and relatively promiscuous modification of the proteome in either human cells or mouse cells deficient in USP18^41,43,62^. However, these studies relied on ectopic expression of ISG15, its E1, E2, and E3 as well as its substrate or treatment with interferon in tissue culture cells, conditions which may not reflect native induction of ISGylation in the context of infection of the whole organism. Since then, there have not been any large-scale, quantitative ISGylomes published. Here, we contribute a large-scale map of endogenous ISG15 sites in a relevant infection setting in vivo. Furthermore, our strategy allowed us to determine the strict ubiquitylome in liver tissue from *Isg15*^−/−^ animals prior to and following infection with *Listeria monocytogenes* in order to identify the ISGylome. In and of itself, the in vivo ubiquitylome following *Listeria* infection had not yet been mapped and similar to what is observed in cells *Listeria* provokes a downregulation of many ubiquitylated targets^46^. In addition, our data confirmed the existence of mixed ISG15-ubiquitin chains in vivo on K29 of ubiquitin as previously reported and potentially on K6, which had not yet been observed^53^. Our analysis also revealed sites of possible ISG15 chains, the function of which remains to be explored.

Our method led to the identification of 614 ISG15 sites on 292 proteins following infection in wild-type animals and 316 ISG15 sites on 219 proteins in USP18^C61A/C61A^ animals, out of ~5000 total identified sites (18.6% of sites following *Listeria* infection). This percentage indicates that ISG15 is a less abundant modification than SUMO or ubiquitin, which is in line with our previous findings^13^. However, this number of sites also implies that some previously identified ubiquitin sites may in fact be misidentified ISG15 sites. Following in vitro viral infection, interferon treatment or overexpression of ISG15, along with its E1, E2, E3, and a target, cotranslational modification has been reported^29^. Our data suggest that during *Listeria* infection in vivo, modification of infection-induced proteins constitutes around 12% of modified proteins during wild-type infection (from cluster 2) and 18% during enhanced ISGylation (cluster 1) by contrast with 25% of ubiquitin substrates (proteins modified that are also upregulated by infection, cluster 3). It will be informative to assess whether viral infection causes a higher rate of infection-induced protein modification than bacterial infection or whether this phenomenon varies from one tissue or cell type to another. Our approach took advantage of *Isg15*-deficient animals, however, one potential caveat could be that the lack of ISG15 provokes compensatory changes in ubiquitin sites or levels. We attempted to assess this possibility with SDS-PAGE and quantitative proteomics of diglycine sites prior to infection and did not find substantive differences between *Isg15*^−/−^ and wild-type. However, using newly available methods such as UbiSite to assess ubiquitin levels in *Isg15*^−/−^ animals or cells would definitively address this caveat^45^. Recently, an elegant biochemical study characterizing a viral effector called LB(Pro) from foot-and-mouth-disease-virus revealed its function as an ISG15 isopeptidase which can also be exploited to identify ISG15 sites^63^. One potential challenge of using a viral protein to identify sites lies in controlling and demonstrating its specificity for ISG15 or ubiquitin, thus future studies comparing substrates from each method would be timely and relevant to identify a core list of ISG15 sites as well as sites that could potentially be unique to specific biological conditions or infections.

This data set reveals many unexpected findings while also confirming previously described observations. If any cellular compartment is particularly enriched across all ISG15 clusters, it is extracellular or secreted proteins, which supports the role of ISG15 in exosome secretion, though TSG101 was not specifically identified as a substrate following *Listeria* infection^32^. USP18 is concomitantly upregulated with ISG15 following *Listeria* infection and whether this is to temporally regulate specific targets or to alter STAT signaling remains to be determined. Therefore, the USP18^C61A/C61A^ ISGylome could potentially represent a distinct subset of ISG15 substrates, which could hypothetically need to be more rapidly removed during the course of an infection for proper antibacterial immunity. Thus, cluster 2 (proteins present in wild-type, but absent in *Isg15*^−/−^ following infection) is likely the closest approximation of the wild-type ISGylome following *Listeria* infection. Cluster 2 displays increased overlap with known acetylation sites, as well as modification of mitochondrial proteins. Protein acetylation in the nucleus, cytosol, and mitochondria controls a variety of cellular functions from gene expression to metabolic pathways and can be modulated by the NAD + -dependent protein deacetylases Sirtuin1-7. Previous work has highlighted the importance of histone deacetylation by Sirtuin 2 for transcriptional reprogramming of an infected cell^64^. Our current work implies that other Sirtuins could also be important for metabolic reprogramming in concert or in competition with ISGylation. One enigmatic finding from the ISG15 literature is the presence of ISGylated conjugates within the mitochondria as determined by protection of ISG15 chains from proteinase K digestion^52^. At least some of the sites on mitochondrial proteins that we identified are located within the inner mitochondrial membrane, and how this occurs topologically remains to be elucidated.

The gene ontology analysis also unearthed enrichment in cofactor and nucleotide-binding proteins and a variety of metabolic pathways. Since we did not find a particular ISG15 motif in either cluster, we analyzed the topography of solved protein structures surrounding the modified lysine. In several cases, sites of dimerization were targeted by ISG15, as were active sites of enzymes. The liver is a site of immune surveillance, metabolic activity, and detoxification of blood, therefore ISGylation may be a unique modification that transmits an alarm signaled by bacterial or viral infection and has metabolic consequences. If modification at sites of dimerization or active sites of metabolic enzymes does reduce the activity of these enzymes, it could be another tool in the arsenal against pathogens that otherwise could usurp cellular metabolites for their own survival and replication. We were intrigued by the modification of the autophagy modulators mTOR, WIPI2, AMBRA1, and RAB7 because modification of mTOR in the FKBP/Rapamycin-binding domain could act to repress its function in growth and metabolism while upregulating the process of xenophagy. The effect of the K2066R mTOR mutant on autophagy induction would support this hypothesis, however, as previously mentioned, this effect was observed in the presence of wild-type mTOR. Future work in a deletion or knockdown background will be even more informative. The consequences of modification sites on WIPI2 and AMBRA1 are more difficult to predict given less is known with regard to their structural features, so we assessed general autophagy levels following infection. Indeed, basal and infection-induced autophagy were increased in USP18^C61A/C61A^ cells and animals relative to wild-type. Previous work has reported modification of the autophagy regulator Beclin-1 by ISGylation^57^, however, this specific modification does not appear to occur during *Listeria* infection. Within cells elevated autophagy led to wild-type or slightly better clearance of bacteria as would be predicted, however, the cell extrinsic role of USP18^C61A/C61A^ mutation in vivo led to a higher bacterial load potentially through the previously reported pleiotropic effects on signaling or cytokine secretion, which could favor *Listeria* replication following unchecked ISGylation. Future work will tackle these discrepancies by assessing the relative contribution of cytokine secretion versus autophagy induction on bacterial burden in vivo. Taken together, we have generated a comprehensive map of ISG15 sites to pave the way to understanding the consequences of ISG15 modification on protein fate and function. In addition, the strategy established here provides a previously unavailable tool to globally identify endogenous and pathogen-derived ISGylation substrates within the context of the whole organism.

## Methods

### Materials

We used an α-ISG15 antibody from Santa Cruz (F-9) at 1:200, an α-SQSTM1/p62 antibody from Abcam, UK (ab56416) at 1:1000. We used an α-LC3 antibody from MBL, Japan (M152-3, clone 4E12) at 1:1000 and an α-ACTIN antibody from Sigma, Saint Louis, MO (AC-15, A5441) at 1:5000. We used an α-mTOR antibody from Sigma (PA5-34663) at 2 µg/sample for immunoprecipitation, an α-mTOR antibody from Cell Signaling (7C10) at 1:1000, an α-tubulin antibody from Sigma (T6074) at 1:5000, an α-ubiquitin antibody from Cell Signaling (P4D1) at 1:1000, and an α-NEDD8 antibody from Cell Signaling (19E3) at 1:1000. We used an α-HA antibody from Sigma (H6908) at 1:1000 for immunoblotting, and an α-GFP antibody from Santa Cruz Biotechnology (sc-81045) at 1:1000. We used an α-FLAG^®^ M2 antibody from Sigma (F3165) at 1:10,000, an α-HA tag antibody—ChIP Grade from Abcam, UK (ab9110) at 1:5000, and an α-alpha-tubulin antibody from Genetex (GT114) at 1:40,000. We used α-HA antibody magnetic beads from Pierce (88837), and anti-flag antibody magnetic beads from Sigma (M2 M8823 Millipore) for immunoprecipitation.

### Bacterial and mammalian growth conditions and infections

*Listeria monocytogenes* strain EGD (BUG 600) was used for all infections in this study. *Listeria* was grown in Brain Heart Infusion media (BD). Prior to infection, overnight cultures of bacterial strains were diluted in new media and grown to exponential phase (OD 0.8–1), washed three times in serum-free mammalian cell culture media, and resuspended in mammalian cell culture media at the indicated MOI. A fixed volume was then added to each well. Cells were centrifuged for 1 min at 201 × *g* to synchronize infection. The cells were then incubated with the bacteria for 1 h at 37 °C, 5% CO_2_. Following this incubation, the cells were washed at room temperature with 1× PBS, and cell growth medium with 10% serum was added with 20 μg/ml gentamicin to kill extracellular bacteria. The cells were then harvested at the indicated time point to collect bacteria for colony-forming units or to collect protein lysates for SDS-PAGE analysis. Mouse embryonic fibroblasts were grown in DMEM with Glutamax (Gibco, Waltham, MA) supplemented with 10% fetal bovine serum. Primary mouse embryonic fibroblasts were transformed through transfection (Fugene HD, Promega) with a plasmid containing the SV40 Large T antigen. Retrovirus was generated by transfection of Phoenix cells (ATCC CRL-3213) with pBabe puro GFP-LC3 (Plasmids listed in Supplementary Data 6) using Fugene HD. In all, 1.5 ml of collected virus was mixed with 500 μl of conditioned media of MEFs supplemented with 8 μg/ml polybrene (Millipore), and applied to MEFs. Virus was collected and applied to MEFs in the presence of polybrene (Millipore) as described (in the protocol “Production of retroviruses using Fugene 6” from the Weinberg lab on Addgene). Cells were then selected using 2 μg/ml puromycin for 3 days. The population of cells that survived puromycin treatment was expanded and tested for GFP-LC3 expression. For maintenance, cells were initially grown without puromycin, however, we observed that the USP18^C61A/C61A^ cells with GFP-LC3 would lose expression over time, likely due to deleterious levels of autophagy, therefore we were required to maintain the cells in puromycin.

### Overexpression systems in tissue culture

Huh-7 cells (obtained from the laboratory of Eliane Meurs at Institut Pasteur) were transfected (Fugene HD, Promega) with 0.5 µg of myc-mTOR or myc-mTOR K2066R. Forty-eight hours post transfection, cells were starved without amino acids with HBSS Ca^2+^ + Mg^2+^ + medium for 4 h. Cells were treated with bafilomycin A1 (100 nM) for 30 min and subsequently lysed with 1× RIPA lysis buffer supplemented with Complete Mini Protease inhibitor cocktail (Roche). HEK293T (obtained from Prof. Sven Eyckerman) or HeLa (obtained from ATCC—CCL2™) cells were grown to ~80% confluence in DMEM + 10% FBS and 1% P/S before being transfected with E1/E2/E3, and HA-ISG15-LRGG or HA-ISG15-LRAA using polyethylenimine (PEI). After 24 h, they were transfected with mGpi1-FLAG, GFP-MYH9, or eGFP-HSP90-β for another 24 h or 48 h. Plasmids used: pcDNA3.1-HA-ISG15, pcDNA3.1-HA-ISG15-LRAA, pcDNA3.1-hUbe1L (E1), pcDNA3.1-UbcM8 (E2), pcDNA3.1-Ubch8 (E2), pTriEx2-hHERC5 (E3), pCMV6-mGpi1-FLAG (Origene), pCMV-GFP-MYH9, pEGFP-HSP90-β (Supplementary Data 6). Cell lines are tested for mycoplasma prior to expansion in the laboratory, and subsequently 2–3 times per year or if they exhibit symptoms (slow growth, increased cell death following transfection or infection). We did not use ICLAC cell lines in this study, but two cell lines (HEK293T and HeLa) were authenticated by PCR single-locus technology by Eurofins on March 20, 2019.

### SDS-PAGE

For the SDS-PAGE analysis of fibroblasts, cells were trypsinized (0.05% trypsin, Invitrogen) and lysed in 1% Triton lysis buffer supplemented with Complete Mini Protease inhibitor cocktail (Roche). Gels were transferred using an iBLOT transfer system (Invitrogen), blocked in 5% milk for 1 hr at RT, incubated with primary antibody overnight at 4 °C, washed with 0.05% Tween in PBS three times (each wash for 7 min), and incubated for 1 h at room temperature with secondary antibody coupled to HRP. Blots were washed again three times with 0.05% Tween in PBS and revealed using ECL Western Blotting Substrate (Pierce, Waltham, MA).

### Immunoprecipitation

Cells were lysed and protein concentration was determined with either a Bradford or BCA assay. In total, 2 mg of total protein were used for either FLAG-, GFP-, or HA-immunoprecipitation, using FLAG-M2 Affinity gel (Merck), µMACS HA isolation kit (Milteny Biotec) or Anti-HA magnetic beads (Pierce) and GFP-Trap^®^ (ChromoTek) according to the manufacturer’s instructions. We expanded mouse embryonic fibroblasts to 12 75-cm^2^ dishes per genotype. Six dishes of each genotype were treated with recombinant mouse type I interferon IFN-alpha A protein (R&D system) at 1000 U/ml for 24 h, and the other six dishes were untreated. At 24 h, posttreatment cells were lysed in 6 ml of RIPA lysis buffer per 75-cm^2^ dish. The lysates from six dishes were combined for each condition, treated with universal nuclease for 2 h at 4 °C (Pierce) and centrifuged. The pellet was discarded, and the protein concentration in the lysate was measured using a BCA assay (Pierce). In all, 1 mg of protein from each condition was used for subsequent immunoprecipitation. Each sample was incubated with 2 µg of mTOR antibody at 4 °C for 72 h. Antibody-mixed samples were then incubated with 40 µl slurry of protein A/G magnetic agarose beads (Thermo Scientific) at 4 °C for 3 h. Beads were pulled down with a magnet (Dyna Mag2, Invitrogen) and washed three times with cold 1× PBS. Proteins were eluted from beads with 0.1 M glycine, pH 2.5, and immediately neutralized with alkaline neutralization buffer, pH 8.0. Eluates were reduced with 1x sample buffer supplemented with 10% β-mecaptoethanol and heated at 95 °C for 5 min.

### Microscopy and image processing

Cells were plated on coverslips the day prior to an experiment. Cells were fixed in 4% PFA (Electron Microscopy Sciences, Hatfield, Pennsylvania) in PBS for 15 min at room temperature. Coverslips were mounted using ProLong Diamond antifade mounting media (Thermo Fisher Scientific, USA), and images were acquired using an inverted wide-field fluorescence microscope (Axio Observer 7, Carl Zeiss Microscopy, Germany) equipped with an Axiocam 506 mono camera and the software ZEN 2.3 Pro. Images were processed using ImageJ^65,66^ in 16-bit and were auto-contrasted with Renyi Entropy threshold preset for optimal resolution. Puncta were counted using the particle analyze function (size threshold: 1–12 pixels). LC3 puncta were enumerated and divided per nuclei within each field. At least 100 cells were counted per experiment, and the data were compiled from three independent experiments. Statistical analysis was conducted using one-way ANOVA and Tukey’s test post hoc.

### In vivo infections

Female C57BL/6 mice (*Usp18*^+/+^ or USP18^C61A/C61A^) were infected intravenously between 8 and 12 weeks of age with 5 × 10^5^ bacteria per animal and sacrificed 72 h following infection. Colony-forming units per organ (liver or spleen) were enumerated after tissue dissociation and serial dilutions in sterile saline. For the SDS-PAGE of animal samples, wild-type mice were infected intravenously with *Listeria* or injected with sterile saline solution, and sacrificed after 72 h. The liver and spleen were isolated and dissociated. Tissue homogenates were then centrifuged at 19,283 × *g* for 10 min at 4 °C. For liver tissue, an aliquot of the soluble fraction below the layer of fat was removed and resuspended in 2x Laemmli buffer. The samples were then run on an SDS-PAGE gel and blotted for ISG15 and actin levels.

### Statistical methods

To determine statistical significance in animal experiments, nonparametric analyses were used since the *n* is too low to determine normal distribution. We used a two-tailed Mann–Whitney test, exact *p-*values are displayed. For enumeration of GFP-LC3 punta and in vitro infection assays, we used one-way ANOVA followed by Tukey’s test post hoc. The statistical methods for the proteomics analysis are discussed in the proteomics methods.

### Ethics statement

This study was carried out in strict accordance with the French national and European laws and conformed to the Council Directive on the approximation of laws, regulations, and administrative provisions of the Member States regarding the protection of animals used for experimental and other scientific purposes (86/609/Eec). Experiments that relied on laboratory animals were performed in strict accordance with the Institut Pasteur’s regulations for animal care and use protocol, which was approved by the Animal Experiment Committee of the Institut Pasteur (approval number no. 03–49).

### Proteomics sample preparation

Three C57BL6/J mice per genotype and per condition (*Isg15*+/+, *Isg15*^−/−^, and USP18^C61A/C61A^) were injected intravenously with 5 × 10^5^ *Listeria monocytogenes* strain EGD or PBS (carrier control), and were monitored and weighed daily. Seventy-two hours post infection, animals were sacrificed, and whole livers were collected and flash-frozen in liquid nitrogen. The livers were homogenized in 10 ml urea lysis buffer containing 9 M urea, 20 mM HEPES pH 8.0, 1 mM sodium orthovanadate, 2.5 mM sodium pyrophosphate, 1 mM β-glycerophosphate. Samples were sonicated by three pulses of 10 s at an amplitude of 20% and centrifuged for 15 min at 16,000 × *g* at room temperature to remove insoluble components. The protein concentration in the supernatants of each replicate was measured using a Bradford assay (Biorad) and equal protein amounts, each containing 10 mg total protein, were used for further analysis. Proteins in each sample were reduced by adding 5 mM DTT and incubation for 30 min at 55 °C. Alkylation of the proteins was done by addition of 10 mM chloroacetamide for 15 min at room temperature in the dark. The samples were diluted with 20 mM HEPES pH 8.0 to a urea concentration of 2 M, and the proteins were digested with 100 µg trypsin (Promega) (1/200, w/w) overnight at 37 °C. Immunocapture of GG-modified peptides was then performed using the PTMScan^®^ Ubiquitin Remnant Motif (K-ε-GG) Kit (Cell Signaling Technology) according to the manufacturer’s instructions. Briefly, peptides were purified on Sep-Pak C18 cartridges (Waters), lyophilized for 2 days, and re-dissolved in 1.4 ml 1x immunoprecipitation buffer supplied with the kit. Note that at this point, aliquots corresponding to 100 µg of digested protein material were taken for shotgun proteomics analysis. Peptides were incubated with the antibody-bead slurry for 2 h on a rotator at 4 °C and after several wash steps, GG-modified peptides were eluted in 100 µl 0.15% TFA and desalted on reversed phase C18 OMIX tips (Agilent), all according to the manufacturer’s protocol. Purified GG-modified peptides were dried under vacuum in HPLC inserts, and stored at −20 °C until LC-MS/MS analysis.

### LC-MS/MS and data analysis

Purified GG-modified peptides were re-dissolved in 20 µl loading solvent A (0.1% TFA in water/ACN (98:2, v/v)) of which 8 µl was injected for LC-MS/MS analysis on an Ultimate 3000 RSLCnano system in-line connected to a Q Exactive HF mass spectrometer (Thermo). Trapping was performed at 10 μl/min for 4 min in loading solvent A on a 20 mm trapping column (made in-house, 100-μm internal diameter (I.D.), 5-μm beads, C18 Reprosil-HD, Dr. Maisch, Germany) and the sample was loaded on a 400 -mm analytical column (made in-house, 75 µm I.D., 1.9 -µm beads C18 Reprosil-HD, Dr. Maisch). Peptides were eluted by a nonlinear increase from 2 to 56% MS solvent B (0.1% FA in water/acetonitrile (2:8, v/v)) over 140 min at a constant flow rate of 250 nl/min, followed by a 15-min wash reaching 99% MS solvent B and re-equilibration with MS solvent A (0.1% FA in water/acetonitrile (98:2, v/v)). The column temperature was kept constant at 50 °C in a column oven (CoControl 3.3.05, Sonation). The mass spectrometer was operated in data-dependent mode, automatically switching between MS and MS/MS acquisition for the 16 most abundant ion peaks per MS spectrum. Full-scan MS spectra (375–1500 m/z) were acquired at a resolution of 60,000 in the orbitrap analyzer after accumulation to a target value of 3,000,000. The 16 most intense ions above a threshold value of 13,000 were isolated for fragmentation at a normalized collision energy of 32% after filling the trap at a target value of 100,000 for maximum 80 ms. MS/MS spectra (200–2000 m/z) were acquired at a resolution of 15,000 in the orbitrap analyzer. From the aliquots for shotgun proteomics analysis, ~3 µg of peptides were injected on the same LC-MS/MS system, using similar settings as described above. Here, the 16 most intense ions above a threshold value of 22,000 were isolated for fragmentation after filling the trap at a target value of 100,000 for maximum 45 ms.

Data analysis was performed with MaxQuant (version 1.5.4.1) using the Andromeda search engine with default search settings including a false discovery rate set at 1% on both the peptide and protein level. Two different searches were performed to analyze the spectra from the GG-enriched samples and the shotgun samples. In both searches, spectra were interrogated against mouse proteins in the Uniprot/Swiss-Prot database (database release version of May 2016 containing 16,789 mouse protein sequences, [www.uniprot.org]) as well as proteins from *L. monocytogenes* strain EGD (Taxonomy ID 1334565, containing 2806 *Listeria* sequences, [http://www.ncbi.nlm.nih.gov/]) with a mass tolerance for precursor ions of 4.5 ppm, and a mass tolerance for fragment ions of 20 ppm and 0.5 Da for the GG-enriched and the shotgun samples, respectively. Enzyme specificity was set as C-terminal to arginine and lysine (trypsin), also allowing cleavage at arginine/lysine–proline bonds with a maximum of three missed cleavages for the GG-enriched samples and a maximum of two missed cleavages for the shotgun samples. Carbamidomethylation of cysteine residues was set as a fixed modification in both searches, and variable modifications were set to oxidation of methionine (to sulfoxides) and acetylation of protein N-termini. The minimum score for modified peptides was set to 30 and for the GG-enriched samples, GlyGly modification of lysine residues was set as an additional variable modification. In both searches, matching between runs was enabled with an alignment time window of 20 min and a matching time window of 1 min. From the shotgun analysis, only proteins with at least one unique or razor peptide were retained leading to the identification of 4146 mouse proteins (listed in the proteinGroups table), while from the GG-enriched samples 5461 potential ISG15 or ubiquitin sites (listed in the GlyGly (K) Sites table) were identified.

Further data analysis was performed with the Perseus software (version 1.5.5.3) after loading the GlyGly (K)Sites table from MaxQuant. Reverse database hits were removed, as well as potential contaminants. The site table was expanded, intensities were log2 transformed and normalized for each sample by subtracting the median intensity. Replicate samples were grouped, sites with less than three valid values in at least one group were removed, and missing values were imputed from a normal distribution around the detection limit. To reveal sites that were significantly regulated, sample groups were defined based on infection (uninfected vs. EGD) and genotype (WT vs. *Isg15*^−/−^ vs. USP18^C61A/C61A^) and a two-way ANOVA test was performed to compare the intensities of the sites in the infection group with the genotype group. For each modification site, this test calculated a *p*-value (actually −log *p*-value) for infection and a *p*-value for genotype. In total, 2166 sites with a *p*-value < 0.01 in at least one of both groups were considered to be significantly regulated. The intensities of these sites are further shown in a heatmap in Fig. 2a after non-supervised hierarchical clustering. The significantly regulated modification sites are reported in the Supplementary Data 1. To reveal ubiquitin sites that were regulated during infection, a *t* test (FDR = 0.05 and S0 = 1) was performed only comparing GlyGly(K) sites in samples from *Isg15*^−/−^ mice. Quantified GlyGly(K) sites (*n* = 3055) and the results of the *t* tests are shown in Fig. 3a, and are listed in Supplementary Data 5. For the analysis of the shotgun data, the proteinGroups table from MaxQuant was loaded in Perseus, reverse database hits were removed as well as proteins only identified by sites. The LFQ intensities were log2 transformed and normalized for each sample by subtracting the median LFQ intensity. Replicate samples were grouped, proteins with less than three valid values in at least one group were removed and missing values were imputed from a normal distribution around the detection limit. To reveal the proteins that were significantly regulated, samples were grouped similarly as described above, and a two-way ANOVA test was performed to compare the LFQ intensities of the proteins in the infection group with the genotype group. In total, 1725 proteins with a *p*-value < 0.01 in at least one of both groups were considered to be significantly regulated. The LFQ intensities of these proteins are further shown in a heatmap in Supplementary Fig. 2c after non-supervised hierarchical clustering. The significantly regulated proteins are reported in the Supplementary Data 3. Since only two major clusters were observed in the heatmap, a *t* test was performed (FDR = 0.05 and S0 = 1) to compare protein intensities between all infected and all noninfected samples. Quantified proteins (*n* = 3055) and the results of the *t* test are listed in Supplementary Data 4 and shown in the volcano plot in Fig. 3a. The significantly up- and downregulated proteins from this test were used to calculate the percentage of sites overlapping with known ubiquitination or acetylation sites in Fig. 2c. GO terms enrichment analyses were performed using the Gene Ontology consortium bioinformatics resources^67,68^. All mass spectrometry proteomics data have been deposited to the ProteomeXchange Consortium via the PRIDE partner repository with the data set identifier PXD011513. The project name is “Proteomics-based identification of ISG15 modification sites during Listeria monocytogenes infection.”

### Sequence conservation analysis

Ortholog sequences for each protein was retrieved from MetaPhOrs^69^ by the application programming interface (API) access using “dbClient” module in python [https://github.com/lpryszcz/metaphors_api]. One set of ortholog sequences was compiled for each given UniProt protein ID. Multiple sequence alignment was performed using MAFFT version 7.157b^70^ for each set of ortholog sequences. Sequence conservation was computed by the occurrence frequency of Lys at the site of interest among the given set of ortholog sequences. Names of species with ISGylation was retrieved from NCBI [https://www.ncbi.nlm.nih.gov/gene/?term=ISG15].

### Structural analysis of available structures

Structure for each protein was retrieved from Protein Data Bank (PDB) using the “uniprot” module [https://pypi.org/project/uniprot/] in python. If there were multiple structures available for a given protein, all structures would be retrieved and analyzed. The solvent accessibility of each site of interest was computed using DSSP [http://www.cmbi.ru.nl/dssp.html]^71^. The relative solvent accessibility (RSA) was then computed by normalizing the solvent accessibility to the maximum allowed solvent accessibility reported in ref. ^72^. All protein structures were rendered and visualized in PyMOL [https://pymol.org].

### Reporting summary

Further information on research design is available in the Nature Research Reporting Summary linked to this article.

## Supplementary information

Supplementary Information

Description of Additional Supplementary Files

Supplementary Data 1

Supplementary Data 2

Supplementary Data 3

Supplementary Data 4

Supplementary Data 5

Supplementary Data 6

Reporting Summary

## Data Availability

We have deposited the proteomic data on the PRIDE database with the data set identifier PXD011513 [http://proteomecentral.proteomexchange.org/cgi/GetDataset?ID=PXD011513]. The project name is “Proteomics-based identification of ISG15 modification sites during *Listeria monocytogenes* infection” and the code has been uploaded to Github (Listeria ISGylome, https://github.com/wchnicholas/Listeria_ISGylome). The source data underlying Figs. 1b–d, 5a–d, f, 6c–e, and Supplementary Figs. 1a–e, 4a, and 5a–g are provided as a Source Data file. Compiled experiments are also shown with individual units as circles on each graph, thus primary data is displayed on each figure as well. All other data are available from the corresponding authors upon reasonable request.

## Source data

Source Data

## Code availability

Custom python scripts for analyzing the deep mutational scanning data have been deposited to GitHub: Listeria ISGylome, https://github.com/wchnicholas/Listeria_ISGylome.
