# Supplementary material for: The in vivo ISGylome links ISG15 to metabolic pathways and autophagy upon Listeria monocytogenes infection
Source: Nat Commun. 2019 Nov 26;10:5383. doi: 10.1038/s41467-019-13393-x (PMC6879477; doi:10.1038/s41467-019-13393-x)
Supplement: Supplementary file 1 — Supplementary Information [file 41467_2019_13393_MOESM1_ESM.pdf]

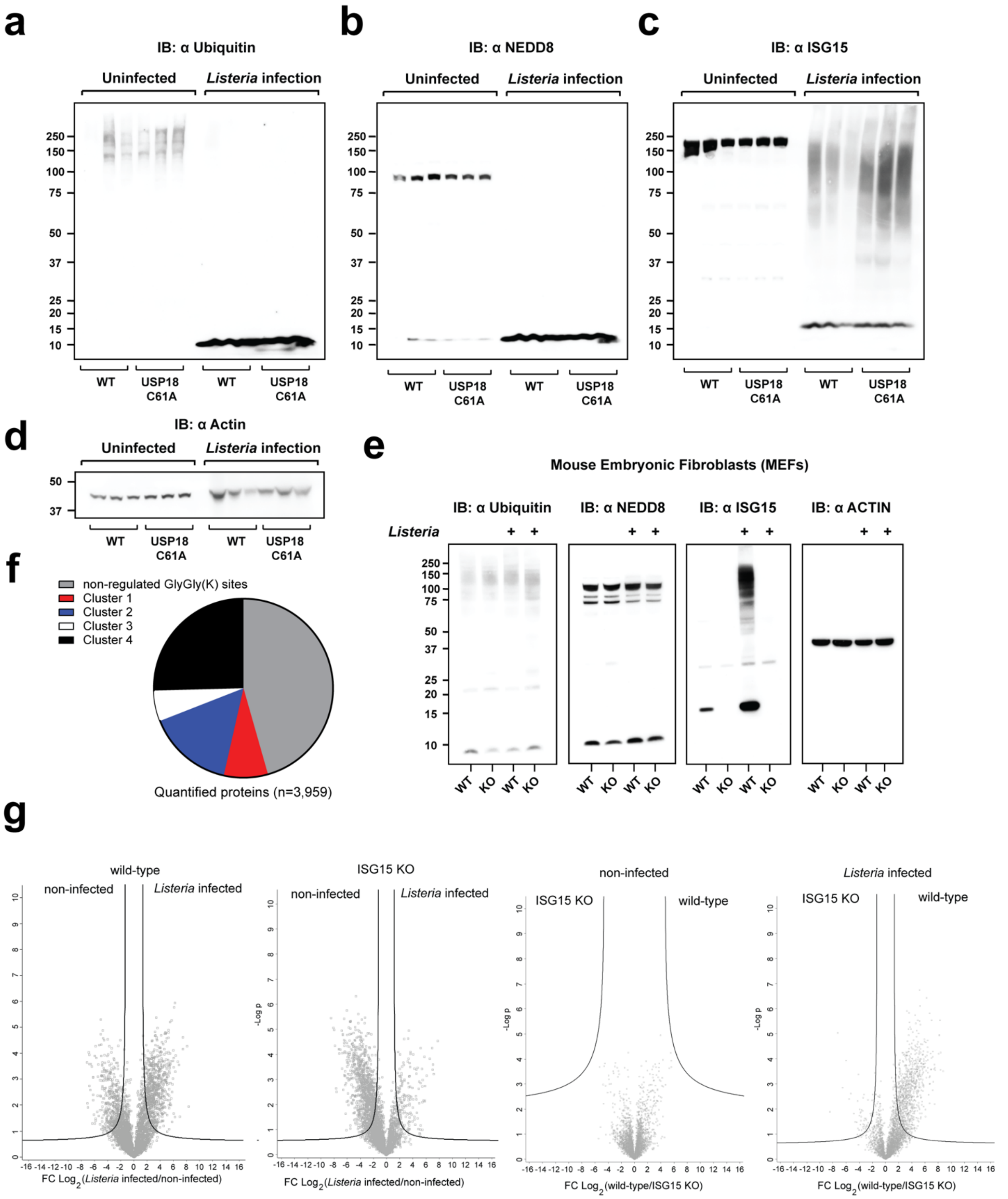

Supplementary Figure 1: Ubiquitin and NEDD8 conjugates decrease following infection with *Listeria* while ISG15 sites increase a-d) SDS-PAGE of liver homogenate from wild type or USP18$^{C61A/C61A}$ mice either uninfected or infected with 5 x 10^5 *Listeria* for 72 hours (three mice of each genotype and condition). e) SDS-PAGE of ubiquitin, NEDD8, ISG15 and ACTIN from MEFs (wild type or *Isg15*$^{-/-}$) uninfected or infected with *Listeria* for 24 hours f) Pie chart showing the distribution of the quantified GlyGly(K) sites between clusters 1 to 4 and the non-significantly regulated sites after two-way ANOVA test (Respectively cluster 1 to 4: 8%, 15.5%, 5.5%, 25.4% and 45.6% of non-significantly regulated sites). g) Volcano plots showing significantly regulated GlyGly(K) sites after pair-wise t-test. The fold change (in log2) of each GlyGly(K) site is shown on the x-axis, while the statistical significance (-log P value) is shown on the y-axis (FDR=0.05, S=1). From left to right: *Listeria* infection upregulated 746 sites in wild type animals while 435 sites were downregulated. In *Isg15*$^{-/-}$ animals *Listeria* upregulated only 168 sites while 1,137 sites were downregulated. No sites were significantly regulated between uninfected wild type and *Isg15*$^{-/-}$ mice. Finally, *Listeria* infection led to 886 upregulated sites in wild type and 54 sites were upregulated in *Isg15*$^{-/-}$ mice. Raw data are available in the source data file.

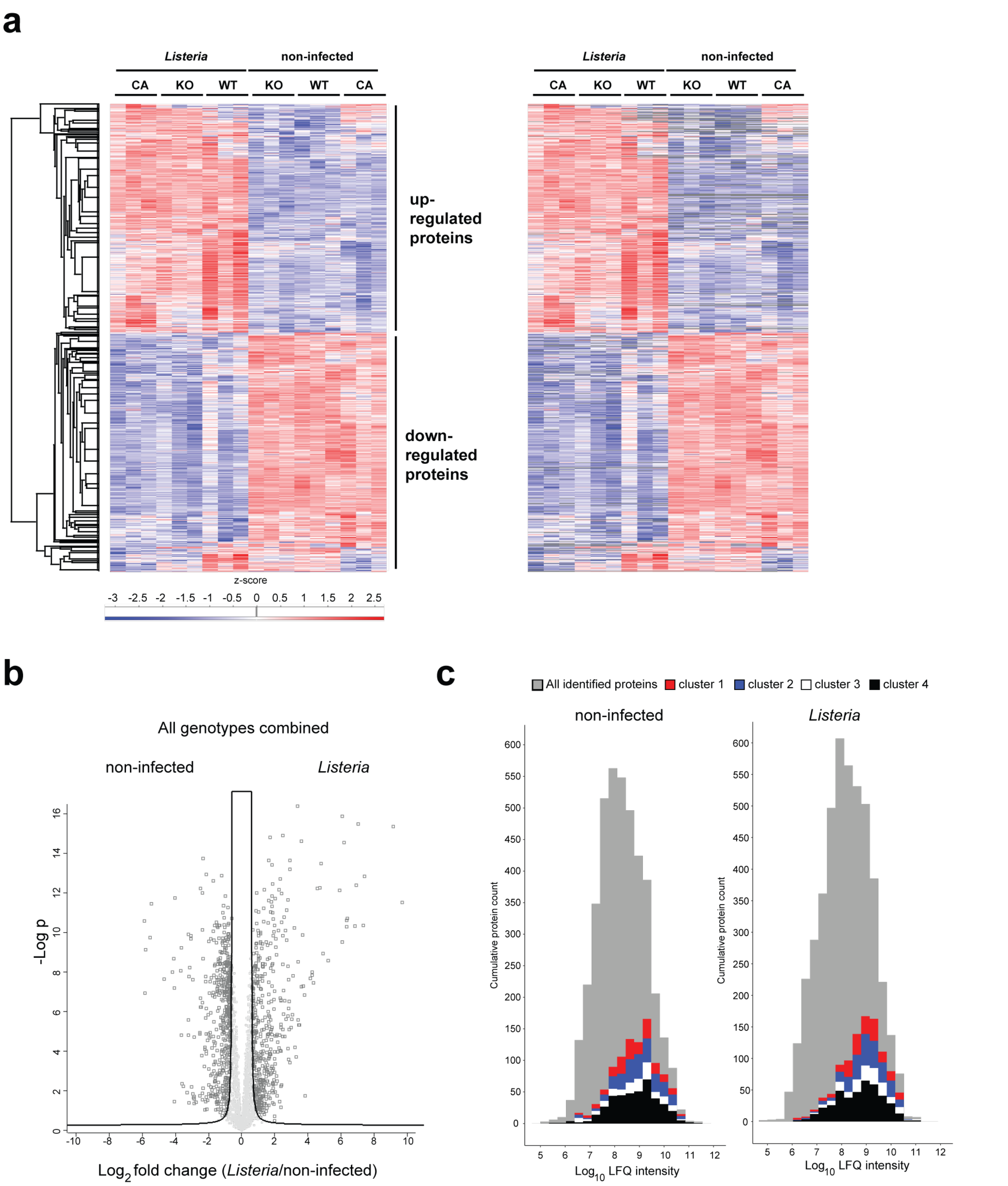

Supplementary Figure 2: Proteomics data for input and ISGylome a) Heatmap showing significantly regulated proteins after non-supervised hierarchical clustering. On the right side, the heatmap is shown with missing values in grey. Two major clusters can be observed corresponding to proteins that are upregulated or downregulated during infection, listed in Supplementary Data 3. b) Since only two major clusters were observed in the heatmap, a t-test was performed (FDR=0.05 and S0=1) to compare protein intensities between all infected and all non-infected samples. Quantified proteins (n=3,055) and the results of the t-tests are listed in Supplementary Data 4. The fold change (in log2) of each protein between the nine infected and nine non-infected samples is shown on the x-axis, while the statistical significance (-log P value) is shown on the y-axis. According to this t-test, 490 proteins were upregulated during infection and 558 were downregulated. c) Distribution of the total proteome, ISGylome (cluster 1 and 2) and ubiquitylome (cluster 3 and 4) in liver from wild type mice infected or not with *Listeria*, based on LFQ intensity (x-axis) and on the cumulative protein counts (y-axis). Although there is a slight bias towards more abundant proteins, proteins at all expression levels were well represented in both the ISGylome and ubiquitinome, independent of infection.

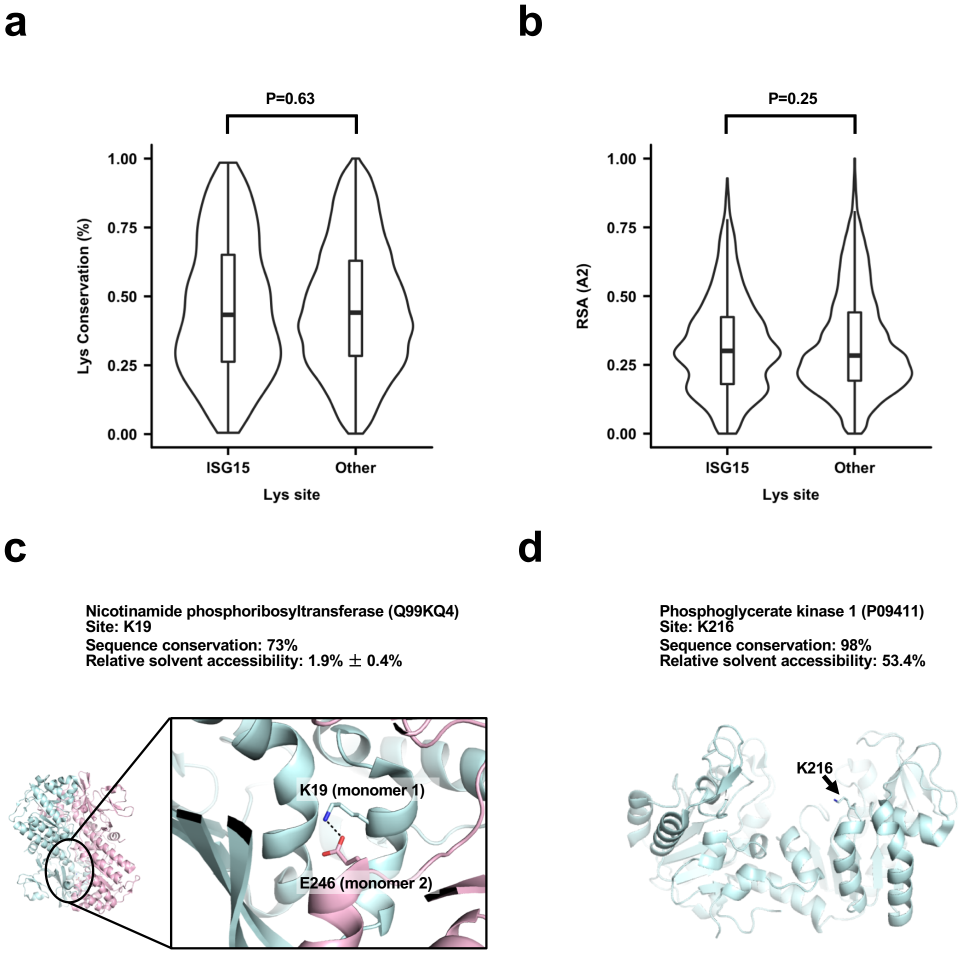

Supplementary Figure 3: General properties of ISGylation sites a-b) All lys residues in the proteins within cluster 1 and 2 were categorized into ISGylated sites ("ISG15") or non-ISGylated sites ("Other"). a) The sequence conservation among orthologs for each Lys site was computed. The distributions of sequence conservation were compared between the two categories. b) The relative solvent accessibility (RSA) for each Lys site was computed if structural information is available. For those Lys sites with multiple structures, the reported RSA was the average among all structures. The distributions of RSA were compared between the two categories. Two-tailed t-test was employed to compute the p-value. c) ISGylated site Lys19 of nicotinamide phosphoribosyltransferase (uniprot ID: Q99KQ4) is shown on the structure (PDB 2H3B)[1]. d) ISGylated site K216 on phosphoglycerate kinase 1 (UniProt ID: P09411) is shown on the structure (PDB: 4O3F)[2].

a

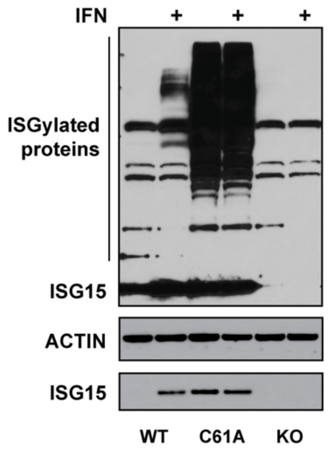

b

Tukey's multiple comparisons test	Mean Diff.	95% CI of diff.	Significant?	Summary	Adjusted P Value
wt uninfected vs. wt un baf	-17.27	-38.09 to 3.558	No	ns	0.2193
wt uninfected vs. wt EGD	-20.24	-37.84 to -2.642	Yes	**	0.0095
wt uninfected vs. wt EGD baf	-31.2	-49.13 to -13.26	Yes	****	< 0.0001
wt uninfected vs. ki uninfected	-31.01	-53.10 to -8.921	Yes	***	0.0003
wt uninfected vs. ki un baf	-42.48	-64.09 to -20.87	Yes	****	< 0.0001
wt uninfected vs. ki EGD	-38.63	-56.63 to -20.63	Yes	****	< 0.0001
wt uninfected vs. ki EGD baf	-56.22	-74.36 to -38.08	Yes	****	< 0.0001
wt uninfected vs. ko uninfected	-17.05	-37.16 to 3.064	No	ns	0.1912
wt uninfected vs. ko un baf	-29.54	-49.94 to -9.148	Yes	***	0.0002
wt uninfected vs. ko EGD	-34.68	-52.68 to -16.68	Yes	****	< 0.0001
wt uninfected vs. ko EGD baf	-40.55	-59.65 to -21.46	Yes	****	< 0.0001
wt un baf vs. wt EGD	-2.976	-20.58 to 14.62	No	ns	> 0.9999
wt un baf vs. wt EGD baf	-13.93	-31.87 to 4.007	No	ns	0.3132
wt un baf vs. ki uninfected	-13.74	-35.83 to 8.345	No	ns	0.6661
wt un baf vs. ki un baf	-25.22	-46.83 to -3.607	Yes	**	0.0078
wt un baf vs. ki EGD	-21.36	-39.36 to -3.360	Yes	**	0.0061
wt un baf vs. ki EGD baf	-38.95	-57.09 to -20.81	Yes	****	< 0.0001
wt un baf vs. ko uninfected	0.2184	-19.89 to 20.33	No	ns	> 0.9999
wt un baf vs. ko un baf	-12.28	-32.67 to 8.118	No	ns	0.7117
wt un baf vs. ko EGD	-17.42	-35.42 to 0.5862	No	ns	0.0687
wt un baf vs. ko EGD baf	-23.29	-42.38 to -4.190	Yes	**	0.004
wt EGD vs. wt EGD baf	-10.95	-25.02 to 3.112	No	ns	0.3089
wt EGD vs. ki uninfected	-10.77	-29.84 to 8.311	No	ns	0.7896
wt EGD vs. ki un baf	-22.24	-40.76 to -3.718	Yes	**	0.0051
wt EGD vs. ki EGD	-18.39	-32.53 to -4.238	Yes	**	0.0014
wt EGD vs. ki EGD baf	-35.98	-50.30 to -21.66	Yes	****	< 0.0001
wt EGD vs. ko uninfected	3.194	-13.56 to 19.94	No	ns	> 0.9999
wt EGD vs. ko un baf	-9.301	-26.39 to 7.789	No	ns	0.8266
wt EGD vs. ko EGD	-14.44	-28.59 to -0.2919	Yes	*	0.0406
wt EGD vs. ko EGD baf	-20.31	-35.83 to -4.794	Yes	**	0.0012
wt EGD baf vs. ki uninfected	0.1877	-19.20 to 19.58	No	ns	> 0.9999
wt EGD baf vs. ki un baf	-11.29	-30.13 to 7.557	No	ns	0.7182
wt EGD baf vs. ki EGD	-7.431	-22.00 to 7.134	No	ns	0.8803
wt EGD baf vs. ki EGD baf	-25.02	-39.76 to -10.29	Yes	****	< 0.0001
wt EGD baf vs. ko uninfected	14.15	-2.956 to 31.25	No	ns	0.2224
wt EGD baf vs. ko un baf	1.653	-15.78 to 19.09	No	ns	> 0.9999
wt EGD baf vs. ko EGD	-3.485	-18.05 to 11.08	No	ns	0.9998
wt EGD baf vs. ko EGD baf	-9.356	-25.25 to 6.542	No	ns	0.7406

Tukey's multiple comparisons test	Mean Diff.	95% CI of diff.	Significant?	Summary	Adjusted P Value
ki uninfected vs. ki un baf	-11.47	-34.31 to 11.36	No	ns	0.8907
ki uninfected vs. ki EGD	-7.619	-27.07 to 11.83	No	ns	0.981
ki uninfected vs. ki EGD baf	-25.21	-44.79 to -5.635	Yes	**	0.0016
ki uninfected vs. ko uninfected	13.96	-7.456 to 35.38	No	ns	0.5963
ki uninfected vs. ko un baf	1.466	-20.22 to 23.15	No	ns	> 0.9999
ki uninfected vs. ko EGD	-3.673	-23.12 to 15.78	No	ns	> 0.9999
ki uninfected vs. ko EGD baf	-9.544	-30.01 to 10.92	No	ns	0.9324
ki un baf vs. ki EGD	3.856	-15.05 to 22.76	No	ns	> 0.9999
ki un baf vs. ki EGD baf	-13.74	-32.77 to 5.300	No	ns	0.4311
ki un baf vs. ko uninfected	25.44	4.511 to 46.36	Yes	**	0.0042
ki un baf vs. ko un baf	12.94	-8.256 to 34.14	No	ns	0.6927
ki un baf vs. ko EGD	7.802	-11.10 to 26.71	No	ns	0.9717
ki un baf vs. ko EGD baf	1.931	-18.02 to 21.88	No	ns	> 0.9999
ki EGD vs. ki EGD baf	-17.59	-32.40 to -2.780	Yes	**	0.006
ki EGD vs. ko uninfected	21.58	4.408 to 38.75	Yes	**	0.0025
ki EGD vs. ko un baf	9.085	-8.418 to 26.59	No	ns	0.8675
ki EGD vs. ko EGD	3.946	-10.70 to 18.59	No	ns	0.9993
ki EGD vs. ko EGD baf	-1.925	-17.90 to 14.05	No	ns	> 0.9999
ki EGD baf vs. ko uninfected	39.17	21.86 to 56.49	Yes	****	< 0.0001
ki EGD baf vs. ko un baf	26.68	9.033 to 44.32	Yes	****	< 0.0001
ki EGD baf vs. ko EGD	21.54	6.726 to 36.35	Yes	***	0.0001
ki EGD baf vs. ko EGD baf	15.67	-0.4579 to 31.79	No	ns	0.066
ko uninfected vs. ko un baf	-12.49	-32.16 to 7.171	No	ns	0.6355
ko uninfected vs. ko EGD	-17.63	-34.80 to -0.4620	Yes	*	0.038
ko uninfected vs. ko EGD baf	-23.5	-41.82 to -5.189	Yes	**	0.0017
ko un baf vs. ko EGD	-5.139	-22.64 to 12.36	No	ns	0.9984
ko un baf vs. ko EGD baf	-11.01	-29.64 to 7.617	No	ns	0.7352
ko EGD vs. ko EGD baf	-5.871	-21.84 to 10.10	No	ns	0.9885

Supplementary Figure 4: ISGylation in WT, USP18$^{C61A/C61A}$ and *Isg15*$^{-/-}$ MEFs at baseline and statistical table for GFP-LC3 enumeration a) SDS-PAGE of wild type, USP18$^{C61A/C61A}$ and *Isg15*$^{-/-}$ MEFs treated with Interferon α (1000 units/mL for 24 hours), bottom ISG15 blot is a short exposure of the upper panel. b) Following one-way ANOVA of GFP-LC3 puncta/cell from Figure 5d we performed Tukey's multiple comparison tests in a pairwise manner between every sample and the complete table of significance and p values are listed above. The exact number of cells counted (from four independent experiments) was the following: wild type untreated n=408; wild type untreated + Bafilomycin A n=450; wild type EGD (*Listeria* infected) n=984; wild type EGD (*Listeria* infected) + Bafilomycin A n=987; USP18$^{C61A/C61A}$ uninfected n=352; USP18$^{C61A/C61A}$ uninfected + Bafilomycin A n=410; USP18$^{C61A/C61A}$ EGD (*Listeria* infected) n=1038; USP18$^{C61A/C61A}$ EGD (*Listeria* infected) + Bafilomycin A n=1014; *Isg15*$^{-/-}$ uninfected n=183; *Isg15*$^{-/-}$ uninfected + Bafilomycin A n=224; *Isg15*$^{-/-}$ EGD (*Listeria* infected) n=710; and *Isg15*$^{-/-}$ EGD (*Listeria* infected) + Bafilomycin A n=470; Raw data are available in the source data file.

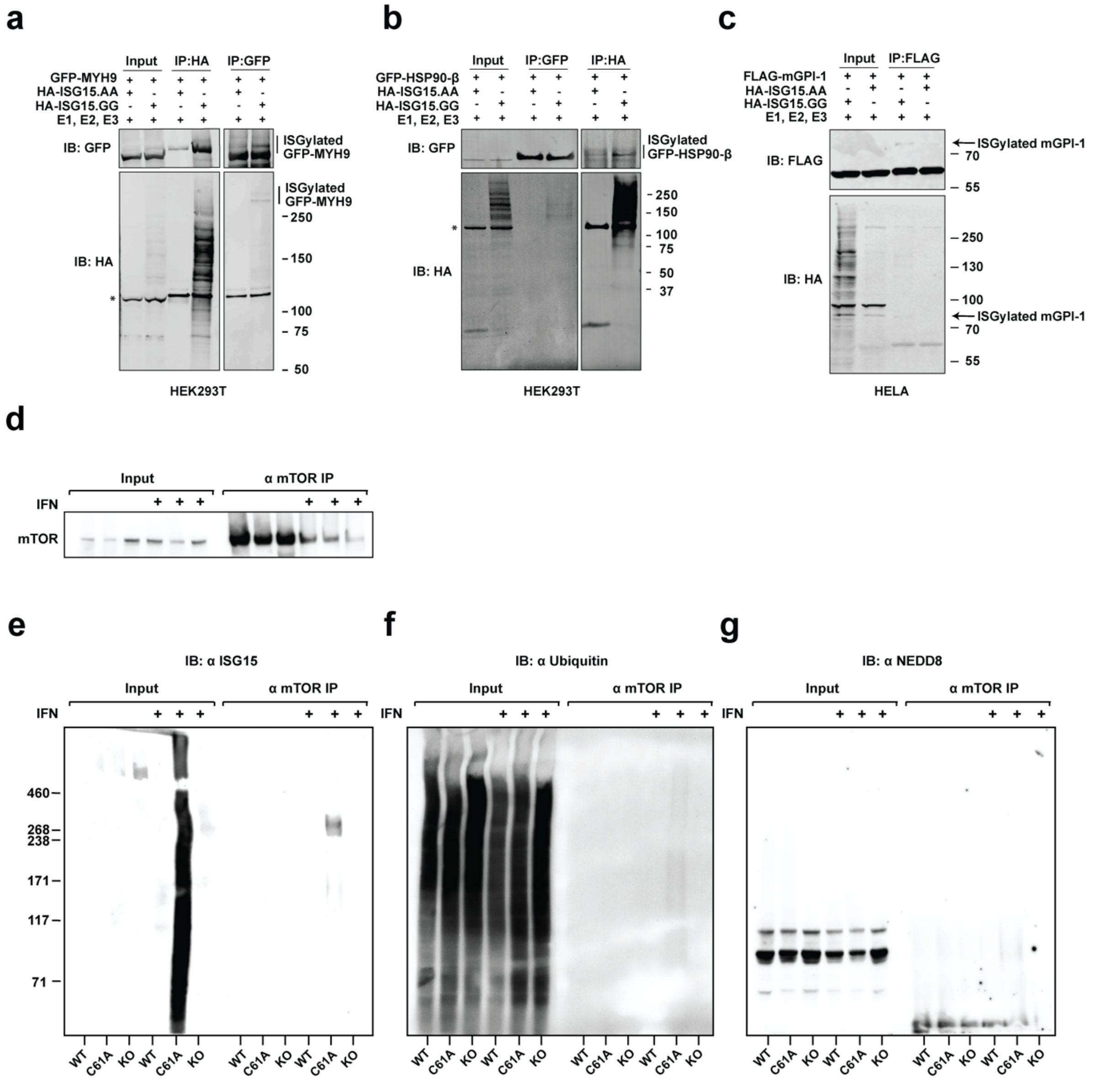

Supplementary Figure 5: Validation of proteins modified by ISG15 a-c) Ectopic expression of ISG15 (either conjugation competent ISG15GG (wild type) or non-conjugatable - ISG15AA) with Ube1L, Ubc8, HERC5 and respective substrates: Myosin 9 and HSP90 in HEK293T or glucose phosphate isomerase in HeLa. Asterisk indicates FLAG-HERC5. d) Immunoblot of mTOR following immunoprecipitation with endogenous mTOR. e-g) Immunoblot of mTOR IP with ISG15, ubiquitin and NEDD8 reveals a more slowly migrating band for ISG15 but not for ubiquitin or NEDD8. Raw data are available in the source data file.

Supplementary References

1. Wang, T. *et al.* Structure of Nampt/PBEF/visfatin, a mammalian NAD+ biosynthetic enzyme. *Nature structural & molecular biology* **13**, 661-662 (2006).

2. Chen, X. *et al.* Terazosin activates Pgk1 and Hsp90 to promote stress resistance. *Nat Chem Biol* **11**, 19-25 (2015).
