# Supplementary material for: The in vivo ISGylome links ISG15 to metabolic pathways and autophagy upon Listeria monocytogenes infection
Source: Nat Commun. 2019 Nov 26;10:5383. doi: 10.1038/s41467-019-13393-x (PMC6879477; doi:10.1038/s41467-019-13393-x)
Supplement: Supplementary file 2 — Description of Additional Supplementary Files [file 41467_2019_13393_MOESM2_ESM.docx]

**Description of Supplementary Files**

**File Name:** Supplementary Data 1

**Description:** List of GlyGly(K) derived from ISG15 (cluster 1 and 2) or ubiquitin (cluster 3 and 4). The GlyGly(K) sites are ordered by cluster ranking according to the heatmap shown in Figure 1. Columns from left to right contain the rank number, the cluster number, the UniProt accession number, the gene name, the protein name, the position of the modified lysine residue in the protein sequence, multiplicity indicates whether the site was found alone (1) or together with other sites on the same peptide (2 or 3), the amino acid sequence surrounding the modified lysine residue (-15; +15) and an indication whether the modified lysine residue was previously reported as ubiquitinated, acetylated or SUMOylated in the PhosphoSitePlus database (version of June 25th 2018, (Hornbeck et al., 2015))49.

**File Name:** Supplementary Data 2

**Description:** List of ISG15 protein substrates (cluster 1 and 2 proteins) in comparison with other studies. Proteins are ordered alphabetically by gene name. Columns from left to right contain the UniProt accession number, the protein name, the gene name and an indication whether the protein was previously reported as an ISG15 protein substrate. The columns L to S list all gene names reported in each study. The gene names specific to human were replaced with their murine orthologues or with the closest murine homologue as annotated in Uniprot database.

**File Name:** Supplementary Data 3

**Description:** List of proteins derived from the protein heatmap obtained after two-way ANOVA and hierarchical clustering. The proteins are ordered by ranking according to the heatmap shown in Figure 3B. Columns from left to right contain the rank number, an indication whether the protein is up or downregulated, the UniProt accession number, the protein name and the gene name.

**File Name:** Supplementary Data 4

**Description:** List of quantified proteins after t-testing. The fold change (in log2) of each protein between the nine infected and nine non-infected samples is reported in column C while the statistical significance (-log P value) is reported in column B. Column A indicates significantly regulated proteins with "+". Column D indicates the UniProt accession number, column E indicates the protein name and column F indicates the gene name. Proteins are also plotted in the volcano plot shown in Figure 3A.

**File Name:** Supplementary Data 5

**Description:** List of quantified GlyGly(K) sites after t-testing. Column C reports the fold change (in log2) of each GlyGly(K) sites between the three infected and three non- infected samples from Isg15-/- samples. Column B reports the statistical significance (-log P value). Column A indicates with "+" whether the GlyGly(K) site is a significantly regulated GlyGly(K) site. Column D contains the UniProt accession number, column E contains the protein name, column F contains the gene name, column G indicates the position of the modified lysine residue in the protein sequence, column H indicates the multiplicity whether the site was found alone (1) or together with other sites on the same peptide (2 or 3), column I contains the amino acid sequence surrounding the modified lysine residue (-15; +15) and column J indicates whether the modified lysine residue was previously reported as ubiquitinated in the PhosphoSitePlus database (version from June 25th 2018, (Hornbeck et al., 2015)49. The GlyGly(K) sites are also plotted in the volcano plot shown in Figure 3A.

**File Name:** Supplementary Data 6

**Description:** List of plasmids used in this study with sequences for unpublished plasmids.
