# Supplementary material for: The in vivo ISGylome links ISG15 to metabolic pathways and autophagy upon Listeria monocytogenes infection
Source: Nat Commun. 2019 Nov 26;10:5383. doi: 10.1038/s41467-019-13393-x (PMC6879477; doi:10.1038/s41467-019-13393-x)
Supplement: Supplementary file 9 — Reporting Summary [file 41467_2019_13393_MOESM9_ESM.pdf]

nature research

Corresponding author(s): Lilliana Radoshevich, Francis Impens and Pascale Cossart

Last updated by author(s): 10/30/2019

Reporting Summary

Nature Research wishes to improve the reproducibility of the work that we publish. This form provides structure for consistency and transparency in reporting. For further information on Nature Research policies, seeAuthors & Referees and theEditorial Policy Checklist .

Statistics

For all statistical analyses, confirm that the following items are present in the figure legend, table legend, main text, or Methods section.

n/a	Confirmed	
☐	☒	The exact sample size (*n*) for each experimental group/condition, given as a discrete number and unit of measurement
☐	☒	A statement on whether measurements were taken from distinct samples or whether the same sample was measured repeatedly
☐	☒	The statistical test(s) used AND whether they are one- or two-sided<br>*Only common tests should be described solely by name; describe more complex techniques in the Methods section.*
☒	☐	A description of all covariates tested
☒	☐	A description of any assumptions or corrections, such as tests of normality and adjustment for multiple comparisons
☐	☒	A full description of the statistical parameters including central tendency (e.g. means) or other basic estimates (e.g. regression coefficient) AND variation (e.g. standard deviation) or associated estimates of uncertainty (e.g. confidence intervals)
☐	☒	For null hypothesis testing, the test statistic (e.g. *F*, *t*, *r*) with confidence intervals, effect sizes, degrees of freedom and *P* value noted<br>*Give P values as exact values whenever suitable.*
☒	☐	For Bayesian analysis, information on the choice of priors and Markov chain Monte Carlo settings
☒	☐	For hierarchical and complex designs, identification of the appropriate level for tests and full reporting of outcomes
☒	☐	Estimates of effect sizes (e.g. Cohen's *d*, Pearson's *r*), indicating how they were calculated

Our web collection on statistics for biologists contains articles on many of the points above.

Software and code

Policy information about availability of computer code

Data collection	Structure for each protein was retrieved from Protein Data Bank (PDB) using the “uniprot” module (https://pypi.org/project/uniprot/) in python. If there were multiple structures available for a given protein, all structures would be retrieved and analyzed. The solvent accessibility of each site of interest was computed using DSSP (http://www.cmbi.ru.nl/dssp.html).
Data analysis	Custom python scripts for analyzing the deep mutational scanning data have been deposited to GitHub Listeria ISGylome (https://github.com/wchnicholas/Listeria_ISGylome)

For manuscripts utilizing custom algorithms or software that are central to the research but not yet described in published literature, software must be made available to editors/reviewers. We strongly encourage code deposition in a community repository (e.g. GitHub). See the Nature Research guidelines for submitting code & software for further information.

Data

Policy information about availability of data

All manuscripts must include a data availability statement. This statement should provide the following information, where applicable:

- Accession codes, unique identifiers, or web links for publicly available datasets
- A list of figures that have associated raw data
- A description of any restrictions on data availability

Proteomics data sets have been deposited on the PRIDE database, accession codes are in the methods and data availability statement.

Field-specific reporting

Please select the one below that is the best fit for your research. If you are not sure, read the appropriate sections before making your selection.

- [x] Life sciences
- [ ] Behavioural & social sciences
- [ ] Ecological, evolutionary & environmental sciences

For a reference copy of the document with all sections, see nature.com/documents/nr-reporting-summary-flat.pdf

Life sciences study design

All studies must disclose on these points even when the disclosure is negative.

Sample size	All experiments were conducted a minimum of three times or in at least three individual animals for histology and western blotting of in vivo lysates. Proteomics data was collected in three individual animals per genotype per condition.
Data exclusions	No data points were excluded.
Replication	Experiments were repeated at least three times to be sure that the data shown in the paper is reproducible.
Randomization	Mass spectrometry results were grouped based on non-supervised hierarchical clustering, thus the samples that were the most similar (ie genotype and infection condition) were sorted by the analytic software as opposed to grouped prior to analysis.
Blinding	LC3 puncta were enumerated using ImageJ to avoid manual counting of samples.

Reporting for specific materials, systems and methods

We require information from authors about some types of materials, experimental systems and methods used in many studies. Here, indicate whether each material, system or method listed is relevant to your study. If you are not sure if a list item applies to your research, read the appropriate section before selecting a response.

Materials & experimental systems

n/a	Involved in the study	
☐	☒	Antibodies
☐	☒	Eukaryotic cell lines
☒	☐	Palaeontology
☐	☒	Animals and other organisms
☒	☐	Human research participants
☒	☐	Clinical data

Methods

n/a	Involved in the study	
☒	☐	ChIP-seq
☒	☐	Flow cytometry
☒	☐	MRI-based neuroimaging

Antibodies

Antibodies used	We used an α-ISG15 antibody from Santa Cruz (F-9) at 1:200, an α-SQSTM1/p62 antibody from Abcam, UK (ab56416) at 1:1000. We used an α-LC3 antibody from MBL, Japan (M152-3, clone 4E12) at 1:1000 and an α-ACTIN antibody from Sigma, Saint Louis, Missouri (AC-15, A5441) at 1:5000. We used an α-mTOR antibody from Sigma (PA5-34663) at 2 μg/sample for immunoprecipitation, an α-mTOR antibody from Cell Signaling (7C10) a t 1:1000, an α-tubulin antibody from Sigma (T6074) at 1:5000, an α-ubiquitin antibody from Cell Signaling (P4D1) at 1:1000, and an α-NEDD8 antibody from Cell Signaling (19E3) at 1:1000. We used an α-HA antibody from Sigma (H6908) at 1:1000 for immunoblotting, and an α-GFP antibody from Santa Cruz Biotechnology (sc-81045) at 1:1000. We used an α-FLAG® M2 antibody from Sigma (F3165) at 1:10000, an α-HA tag antibody - ChIP Grade from Abcam, UK (ab9110) at 1:5000, and an α-alpha-Tubulin antibody from Genetex (GT114) at 1:40000. We used α-HA antibody magnetic beads from Pierce (88837), and anti-flag antibody magnetic beads from Sigma (M2 M8823 Millipore) for immunoprecipitation.
Validation	Where possible we use knock out lines to validate antibodies, otherwise we use commercial antibodies cited by reputable authors who are well known in the autophagy field.

Eukaryotic cell lines

Policy information about cell lines

Cell line source(s)	Mouse embryonic fibroblasts were generated from wild type, knock-out animals or KI animals and transformed in my lab using SV40 Large T antigen. We used Huh-7 cells from the Cossart laboratory (original source was Eliane Meurs laboratory at the Pasteur Institute), we used HEK293T (obtained from Prof. Sven Eyckerman) and HeLa CCL2 from ATCC that were both authenticated by Eurofins on March 20, 2019.

Authentication	Cells were initially genotyped using PCR and subsequently validated using western blotting. HeLa and HEK293T were authenticated by PCR single locus technology by Eurofins on March 20, 2019.
Mycoplasma contamination	We consistently and regularly test for mycoplasma using kits and PCR which is even more sensitive. All of our lines are negative.
Commonly misidentified lines (See ICLAC register)	We only used fibroblasts that we generated in my laboratory, HeLa cells from ATCC, Huh7 cells and 293T HEK cells from ATCC, none of these cells lines are listed as commonly misidentified on ICLAC.

Animals and other organisms

Policy information about studies involving animals; ARRIVE guidelines recommended for reporting animal research

Laboratory animals	We used C57BL/J mice that were wild type, deleted for ISG15 and a knock in mutation of USP18 C61A.
Wild animals	n/a
Field-collected samples	*For laboratory work with field-collected samples, describe all relevant parameters such as housing, maintenance, temperature, photoperiod and end-of-experiment protocol OR state that the study did not involve samples collected from the field.*
Ethics oversight	This study was carried out in strict accordance with the French national and European laws and conformed to the Council Directive on the approximation of laws, regulations and administrative provisions of the Member States regarding the protection of animals used for experimental and other scientific purposes (86/609/Eec). Experiments that relied on laboratory animals were performed in strict accordance with the Institut Pasteur's regulations for animal care and use protocol, which was approved by the Animal Experiment Committee of the Institut Pasteur (approval number n°03–49).

Note that full information on the approval of the study protocol must also be provided in the manuscript.
